# Enhancing Generic Reaction Yield Prediction through Reaction Condition-Based Contrastive Learning

**DOI:** 10.34133/research.0292

**Published:** 2024-01-12

**Authors:** Xiaodan Yin, Chang-Yu Hsieh, Xiaorui Wang, Zhenxing Wu, Qing Ye, Honglei Bao, Yafeng Deng, Hongming Chen, Pei Luo, Huanxiang Liu, Tingjun Hou, Xiaojun Yao

**Affiliations:** ^1^ Dr. Neher’s Biophysics Laboratory for Innovative Drug Discovery, State Key Laboratory of Quality Research in Chinese Medicine, Macau Institute for Applied Research in Medicine and Health, Macau University of Science and Technology, Macao 999078, China.; ^2^ Innovation Institute for Artificial Intelligence in Medicine of Zhejiang University, College of Pharmaceutical Sciences, Zhejiang University, Hangzhou 310058, China.; ^3^ Faculty of Applied Sciences, Macao Polytechnic University, Macao 999078, China.; ^4^ CarbonSilicon AI Technology Co. Ltd, Hangzhou, Zhejiang 310018, China.; ^5^ Center of Chemistry and Chemical Biology, Guangzhou Regenerative Medicine and Health Guangdong Laboratory, Guangzhou 510530, China.

## Abstract

Deep learning (DL)-driven efficient synthesis planning may profoundly transform the paradigm for designing novel pharmaceuticals and materials. However, the progress of many DL-assisted synthesis planning (DASP) algorithms has suffered from the lack of reliable automated pathway evaluation tools. As a critical metric for evaluating chemical reactions, accurate prediction of reaction yields helps improve the practicality of DASP algorithms in the real-world scenarios. Currently, accurately predicting yields of interesting reactions still faces numerous challenges, mainly including the absence of high-quality generic reaction yield datasets and robust generic yield predictors. To compensate for the limitations of high-throughput yield datasets, we curated a generic reaction yield dataset containing 12 reaction categories and rich reaction condition information. Subsequently, by utilizing 2 pretraining tasks based on chemical reaction masked language modeling and contrastive learning, we proposed a powerful bidirectional encoder representations from transformers (BERT)-based reaction yield predictor named Egret. It achieved comparable or even superior performance to the best previous models on 4 benchmark datasets and established state-of-the-art performance on the newly curated dataset. We found that reaction-condition-based contrastive learning enhances the model’s sensitivity to reaction conditions, and Egret is capable of capturing subtle differences between reactions involving identical reactants and products but different reaction conditions. Furthermore, we proposed a new scoring function that incorporated Egret into the evaluation of multistep synthesis routes. Test results showed that yield-incorporated scoring facilitated the prioritization of literature-supported high-yield reaction pathways for target molecules. In addition, through meta-learning strategy, we further improved the reliability of the model’s prediction for reaction types with limited data and lower data quality. Our results suggest that Egret holds the potential to become an essential component of the next-generation DASP tools.

## Introduction

Efficient chemical synthesis is crucial to satisfying the future demands for pharmaceuticals, materials, and energy [1]. Corey and Wipke [2] first proposed the concept of computer-aided synthesis planning (CASP) in the 1960s. CASP programs take the target molecule as input and return a series of single-step reactions that decompose the target molecule into a set of commercially available starting compounds or simple precursors that can be easily synthesized [3]. A feasible synthesis plan may dramatically accelerate the synthesis of desired molecules [4]. In recent years, with the development of data science, deep learning (DL) algorithms, and computing power, DL-assisted synthesis planning (DASP) has gained considerable interest [5–17]. Modern DASP programs can quickly plan multiple potential retrosynthetic pathways for a given target molecule according to the constraints set by the user for the retrosynthetic search (such as the overall search time and number of single-step expansion steps) [18]. However, these theoretically feasible reaction pathways often become impractical because of such factors as incomplete conversion of reactants, side reactions, or inadequate purification [19]. Therefore, retrosynthetic route planning is only a major component of a successful DASP system [20]. To provide feasible suggestions that can be implemented by chemists in the laboratory, it is necessary to identify the optimal reaction conditions for the retrosynthetic route [21] and evaluate the quality of the overall synthesis route, and reaction yield is one of the most scientific and intuitive metrics for screening reaction conditions and evaluating synthesis pathway [22,23].

**Fig. 1.**
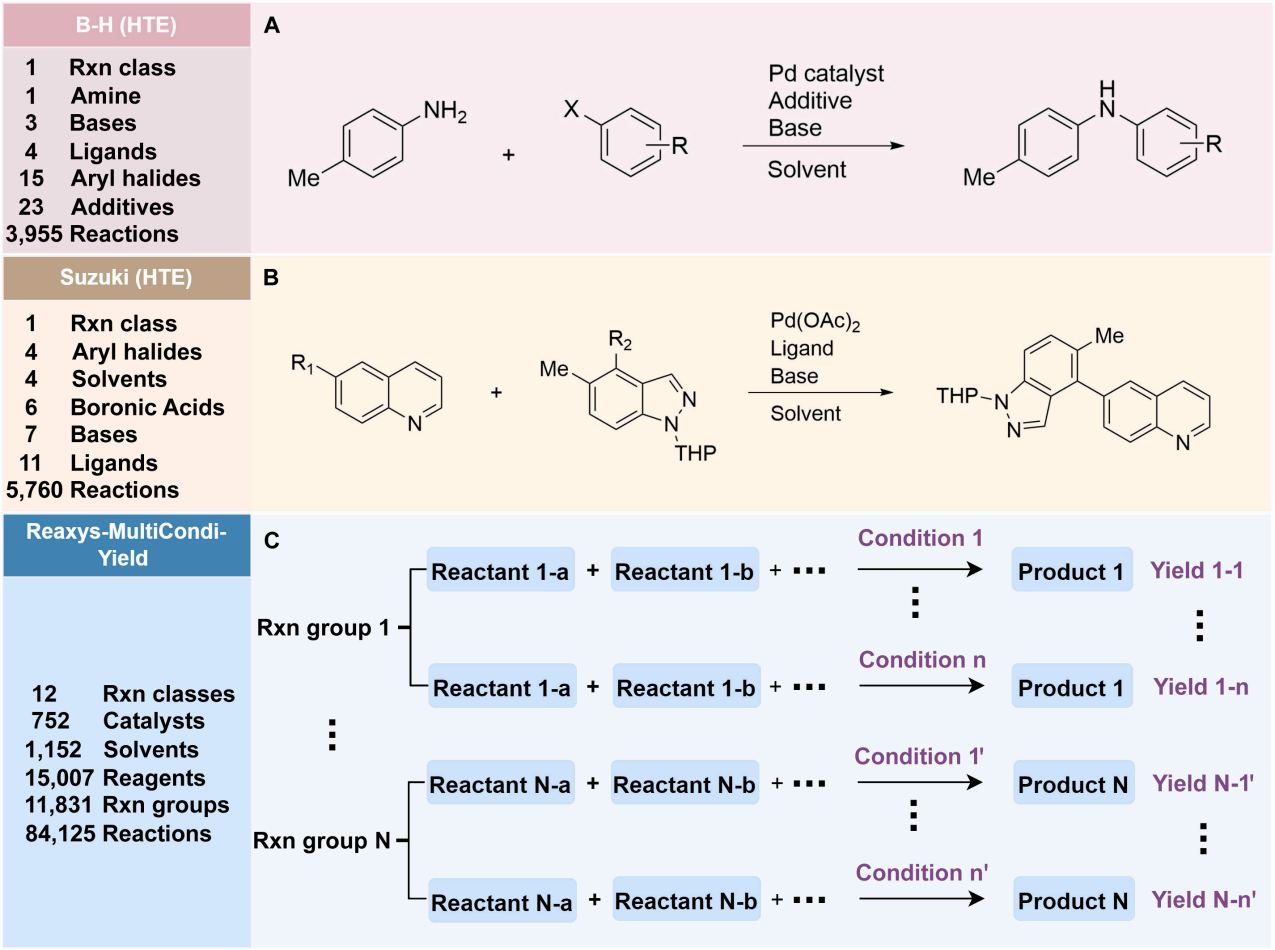
Overall reaction and variables for the Buchwald–Hartwig (B-H) (A), Suzuki–Miyaura (B), and Reaxys-MultiCondi-Yield (C) datasets.

Reaction yield refers to the percentage of reactants that are successfully converted to the desired product [24]. Models that can reliably predict actual yields not only serve as scoring functions of DASP but also help chemists evaluate the overall yield of complex reaction pathways, giving priority to high-yield reactions to save time and cost in wet experiments [25]. However, because of the complexity of molecular structures, the multidimensionality of chemical reactions, and the limited availability of data, it is still a great challenge to predict the yields of chemical reactions under specific conditions [26]. The current yield prediction models are mainly built on high-throughput experimental (HTE) datasets, and Buchwald–Hartwig reactions [26–28] and Suzuki–Miyaura reactions [29,30] are the 2 most well-studied HTE yield datasets (Fig. 1A and B). Early studies utilized computed physicochemical descriptors [26], one-hot encoding of reactions [27], or structure-based molecular fingerprints [28] to predict the yields for these 2 datasets. Recently, the DL fingerprint rxnfp developed by Schwaller et al. [31] and the differential reaction fingerprint drfp developed by Probst et al. [32] have substantially outperformed previous methods. Rxnfp and drfp respectively achieved the best performance on test sets of Buchwald–Hartwig dataset and Suzuki–Miyaura dataset (70:30 random split), with coefficient of determination (*R*^2^) scores of 0.95 and 0.85. However, by studying previous experimental results, we found that most of the aforementioned methods did not achieve the ideal predictive performance on the out-of-sample test sets of Buchwald–Hartwig reactions containing additional additives. This indicates the limitations of using HTE datasets for yield prediction. HTE datasets usually involve specific classes of reactions and focus on a narrow chemical space. When using yield prediction models to explore unknown chemical spaces, this performance degradation problem will be prevalent because the unknown chemical space to be predicted can be very large [33]. Yield prediction models trained on HTE datasets cannot be applied in the real-world scenarios aimed at predicting the yields for a broad variety of reactions. Therefore, curating generic reaction yield datasets that are not limited to specific reaction classes is the first step to promote the practical application of yield prediction models.

Another key point to apply yield prediction models to chemical synthesis practice is to adopt effective modeling methods for generic reaction yield datasets. Reaction Simplified Molecular Input Line Entry System (SMILES) is a simplified chemical language for representing chemical reactions [34]. Therefore, SMILES-based yield prediction can be viewed as a natural language processing (NLP) problem, extracting molecular features directly from reaction SMILES without relying on any manually generated feature. In 2017, Vaswani et al. [35] proposed the transformer architecture for handling various NLP tasks, which achieved excellent feature extraction capability through the self-attention mechanism. In recent years, many pretraining language models such as bidirectional encoder representations from transformers (BERT) [36] and generative pretrained transformer (GPT) [37] based on transformer have been proposed. For example, Schwaller et al. [38] fine-tuned a BERT model pretrained on a large unlabeled reaction corpus on downstream yield prediction tasks and achieved satisfactory performance, demonstrating the advantage of the BERT-based pretraining for chemical reaction yield prediction. In chemical experiments, even for the same set of reactants and target products, the reaction yields may vary widely because of different reaction conditions [39]. Appropriate reaction conditions are key to achieving high-yield chemical reactions [40]. However, because of the limitations of current available reaction yield datasets and modeling methods, existing yield prediction methods have not fully considered the impact of varying reaction conditions on the yields when reactants and products are identical. This leads to the inability of yield prediction models to accurately predict the changes of reaction yields based on different reaction conditions. To address this issue, we can explore the use of contrastive learning strategy [41,42] to enhance the model’s sensitivity to reaction conditions on the specifically curated generic reaction yield dataset with rich reaction condition information. Furthermore, some reaction classes in generic reaction yield datasets may not have sufficient samples. In this regard, meta-learning strategies can be adopted to improve the yield prediction performance under the setting of few-shot learning.

**Fig. 2.**
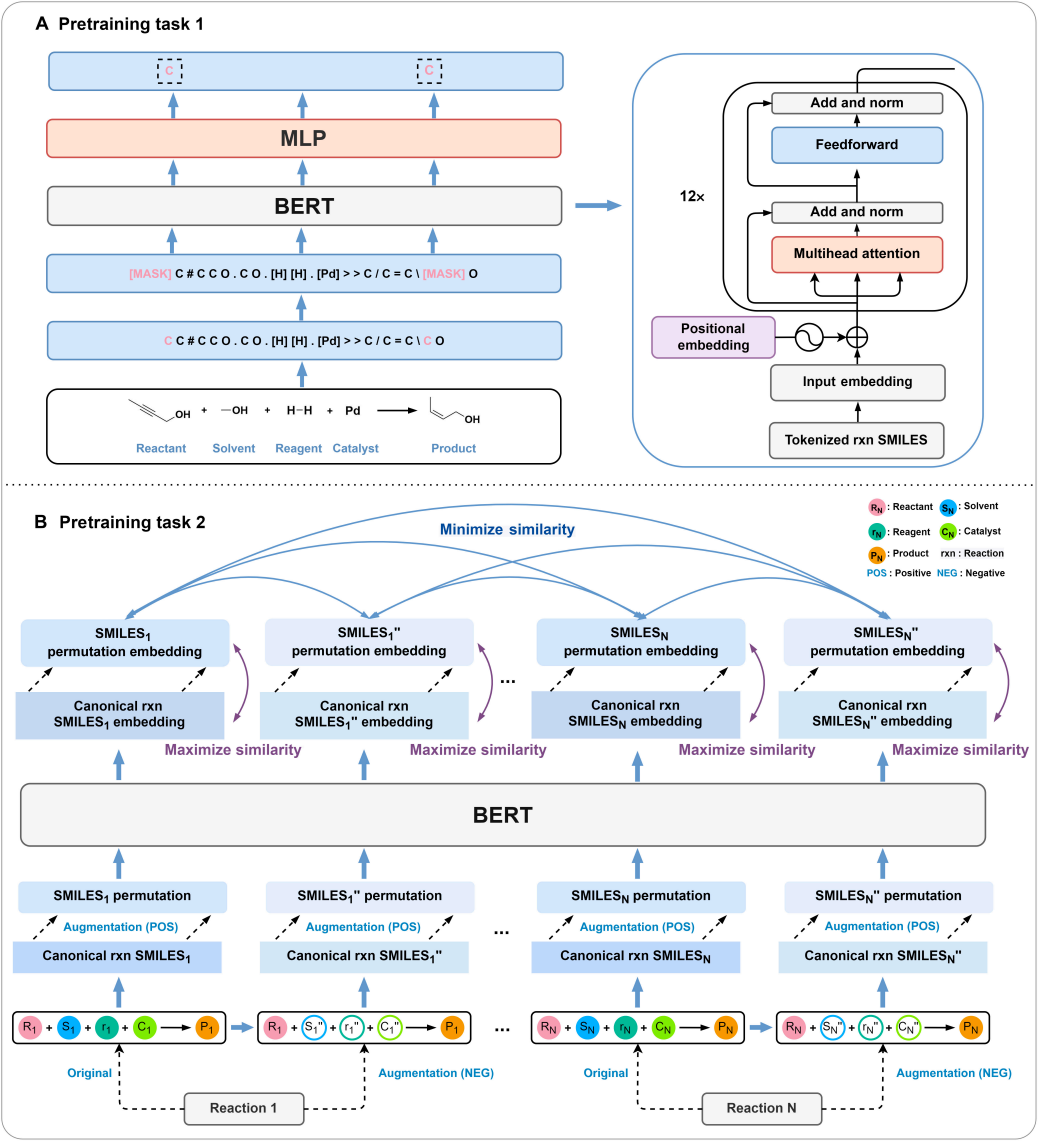
Overview of Egret. (A) Pretraining task 1: MLM task; (B) pretraining task 2: reaction-condition-based contrastive learning task.

The contributions of this work can be briefly summarized as follows:

1. We extracted and curated a high-quality generic reaction yield dataset named Reaxys-MultiCondi-Yield from the Reaxys database (Fig. 1C). Compared to HTE datasets, Reaxys-MultiCondi-Yield encompasses a broader chemical space, including 12 reaction types, 752 catalysts, 1,152 solvents, 15,007 reagents, and 84,125 reactions. Specifically, this dataset consists of 11,831 reaction groups, which are sets of reactions with the same reactants and products but varying yields due to different reaction conditions.
2. To implement a general yield prediction model, we designed a pretraining framework named Egret (Fig. 2), which is based on BERT and includes 2 pretraining tasks: masked language modeling (MLM) and reaction-condition-based contrastive learning. Egret performed comparably or even better than the previous best models on 4 benchmark datasets and achieved optimal performance on the Reaxys-MultiCondi-Yield dataset.
3. We proposed a yield-incorporated scoring for multistep retrosynthesis planning, and the results indicated that the yield-incorporated scoring can indeed prioritize literature-supported high-yield synthesis routes for target molecules.
4. Finally, we used a meta-learning strategy to model the low-sample-size or low-quality data of 5 reaction classes in the Reaxys-MultiCondi-Yield dataset, resulting in a significant improvement in prediction accuracy. Specifically, the accuracy of the reaction class 10 has increased by 33.33%.

## Results

### Dataset

#### Pretraining dataset and benchmark datasets

Reaction data in Pistachio [43] dataset were used for the pretraining of Egret. Here, 4 benchmark datasets were prepared. Among them, Buchwald–Hartwig dataset [26] consists of 3,955 reactions whose reaction space contains 1 amine, 3 bases, 4 ligands, 15 aryl halides, and 23 isoxazole additives (Fig. 1A). Suzuki–Miyaura dataset [44] consists of 5,760 reactions, and its chemical space contains 4 aryl halides, 4 solvents, 6 boronic acids, 7 bases, and 11 ligands (Fig. 1B). US Patent and Trademark Office (USPTO) yield dataset [31] was split into subgram dataset and gram dataset by Schwaller et al. according to product mass. However, these reaction data were originally obtained through text mining from open-access texts from the US Patents database, and it may lack complete experimental details, such as reaction conditions. The data split methods of these 4 benchmark datasets are consistent with previous studies, and we also resplit the 2 USPTO yield datasets according to the ratio of training:validation:test = 6:2:2.

#### Reaxys-MultiCondi-Yield

**Fig. 3.**
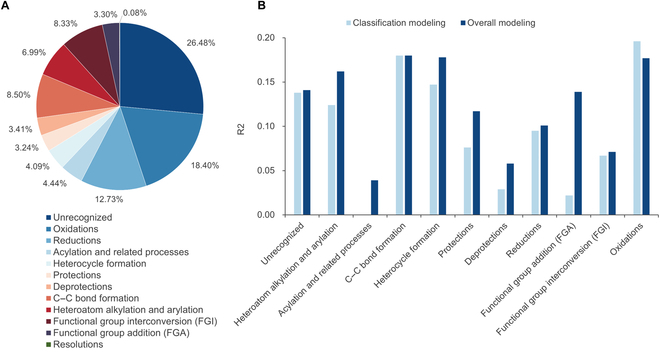
Visualization of the Egret model performance based on modeling strategies. (A) Reaction category composition of the Reaxys-MultiCondi-Yield dataset; (B) prediction performance on the Reaxys-MultiCondi-Yield dataset.

According to the specific data processing procedure that is easy to reproduce (Section S1.2), we exported and curated a generic reaction yield dataset called Reaxys-MultiCondi-Yield from the Reaxys database. Within this dataset, a reaction group refers to a collection of reactions characterized by identical reactants and products but different yields, which result from different reaction conditions (Fig. 1C). The dataset comprises 11,831 reaction groups and 84,125 reactions, and it encompasses a chemical space comprising 15,007 reagents, 1,152 solvents, and 752 catalysts. Moreover, we utilized a neural network classifier based on rxnfp, trained on the Pistachio dataset, to assign reaction categories to the cleaned Reaxys-MultiCondi-Yield dataset, and the accuracy of the reaction classifier on the Pistachio test set achieved 97.8%. As a result, the reaction data in the Reaxys-MultiCondi-Yield dataset were classified into 12 categories. As illustrated in Fig. 3A, aside from the reactions for which the reaction classifier could not predict the type (unrecognized), the most common type of reaction in the Reaxys-MultiCondi-Yield dataset is oxidation, followed by reduction reactions. The remaining types of reactions, in descending order of their proportions, are C–C bond formation, functional group interconversion, heteroatom alkylation and arylation, acylation and related processes, heterocycle formation, deprotections, functional group addition, protections, and resolutions. This demonstrates that the Reaxys-MultiCondi-Yield dataset includes a rich variety of chemical transformations, which is beneficial for developing generalized models for predicting reaction yields. In addition, we further visualized and discussed the distribution and characteristics of the reaction data in the Reaxys-MultiCondi-Yield dataset in Section S1.3. The Reaxys-MultiCondi-Yield dataset was split into the training, validation, and test sets at the 8:1:1 and 6:2:2 ratios using stratified sampling based on reaction groups.

### Model architecture

BERT has achieved impressive successes in various NLP tasks by pretraining the transformer encoder [45–48], and the BERT models trained on chemical reaction data may embed any reaction data (in the form of SMILES) in an abstract vector space, without first preprocessing the reaction data, such as atom mapping. Encouraged by the convenience of such a deep learning paradigm that may autonomously extract and learn useful relations from a large-scale reaction data, we proposed a generic pretrained yield predictor, BERT (Egret; see Fig. 2), which can be broadly applied to any reaction records for yield estimation without being restricted to only some specific reaction classes. Egret consists of 2 pretraining tasks: MLM (task 1) and contrastive learning (task 2).

#### MLM task

The canonical reaction SMILES were tokenized using the procedure proposed by Schwaller et al. [49] for the MLM task. The goal is to train the model to predict individual tokens that have been randomly masked in a given reaction SMILES as shown in Fig. 2A. MLM helps the model to better learn the syntax and semantics of chemical reactions in a self-supervised manner.

#### Contrastive learning task

For a canonical reaction SMILES, a negative reaction SMILES was generated by randomly replacing some of its reaction conditions (reagents, solvents, and catalysts), and then the positive reaction SMILES of the reaction SMILES was generated by SMILES permutation (Fig. 2B), and we have described the process of obtaining the positive and negative reaction samples in detail in Section S1.4. The objective of this task is primarily twofold: (a) to enable the model to distinguish negative reaction samples by minimizing the embedding similarity between reactions involving the same reactants and products but under different conditions, thereby obtaining distinctive feature representations for negative samples; 2) to achieve a robust representation of chemical reactions in the model by maximizing the embedding similarity of different SMILES forms of the same reaction (positive reaction samples), thereby reducing the uncertainty caused by the diversity of reaction SMILES. Therefore, the contrastive learning task uses the following loss function:

$$\begin{array}{c} \text{CL}\;\text{loss}=\alpha \sum_{n=1}^{N}\sum_{i\in I_{n}}\text{cos}\left(V_{n,c},V_{n,i}\right)\\ \;\;+\sum_{n=1}^{N}\sum_{p\in P_{n}}\frac{1}{2}\left(1-\text{cos}\left(V_{n,c},V_{n,p}\right)\right),\alpha \in \left\{0.02,0.05,1\right\} \end{array}$$

$$\cos\left(V_{a}V_{b}\right)=\frac{V_{a}∙V_{b}}{\left\|V_{a}\right\|\left\|V_{b}\right\|}$$

where *n* denotes a reaction record and *N* is the number of reactions in a batch. *I_n_* represents the negative reaction SMILES of reaction *n* in a batch, and *P_n_* represents the positive reaction SMILES of reaction *n*. *V*_*n*, *c*_ is the embedding of the canonical reaction SMILES of reaction *n* generated by Egret, and *V*_*n*, *i*_ and *V*_*n*, *p*_ are the embeddings generated by Egret for the canonical reaction SMILES of reactions *i* and *p*, respectively. *α* is a balance coefficient, and we tested 3 numbers (0.02, 0.05, and 1). When *α* is 0.02, the model converged most stably. cos() denotes the cosine similarity function, which assesses the similarity of 2 embeddings. ‖*V_a_*‖ is the Euclidean norm of the embedding *V_a_* = (*V*_*a*,1_, *V*_*a*,2_, ⋯, *V*_*a*, *p*_), defined as $\sqrt{V_{a,1}^{2}+V_{a,2}^{2}+\cdots +V_{a,p}^{2}}$. In addition, in the second stage of Egret pretraining, the loss includes both the MLM pretraining and the contrastive learning, and the overall loss function reads,

$$\text{Loss}=\sum_{n=1}^{N}\text{CrossEntropyLoss}\left(t_{j},{\hat{t}}_{j}\right)+\mathrm{CL}\;\text{loss}$$

where *t_j_* is the predicted value of the masked token and ${\hat{t}}_{j}$ is the ground truth of the masked token.

### Model performance

#### Benchmark datasets

**Fig. 4.**
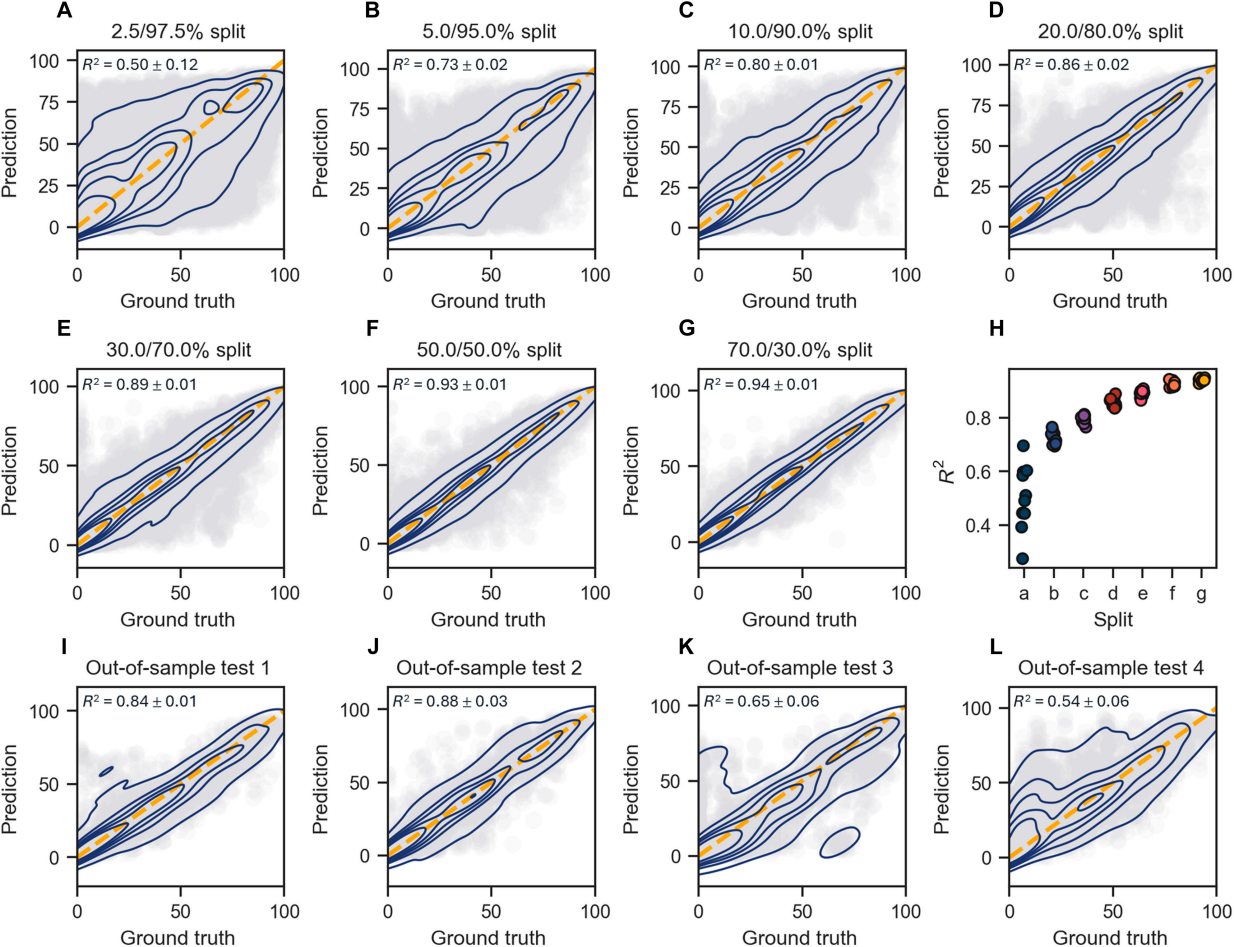
Regression plots of the Buchwald–Hartwig dataset using 11 different data partitioning methods. (A to G) Seven splits with training set proportions ranging from 2.5% to 70%; (I to L) 4 out-of-sample test splits.

For the Buchwald–Hartwig dataset, we tested the performance of Egret on 11 different data partitioning schemes. These schemes included 7 partitions with varying relative sizes of the training set, ranging from 70% down to 2.5%, as well as 4 out-of-sample partitions based on the addition of isoxazole as additives. Each of the first 7 data partitions has 10 random splits, and we optimized the hyperparameters on the training set of the first random split and applied them to the remaining splits. The obtained performance (*R*^2^) was compared with 3 competitive methods: Yield-BERT based on the DL fingerprints [31], XGBoost based on drfp (DRFP) [32], and a classical-descriptor-based method: random forest based on the density functional theory (DFT)-derived fingerprints [26]. The results are shown in Table 1, Egret achieved the best *R*^2^ in 6 of 11 data splits, highlighting the effectiveness of our pretraining strategy. Across the 7 different data partitions with varying proportions of the training set, Egret outperformed DFT and Yield-BERT, while being slightly inferior to DRFP. It is gratifying to note that Egret achieved the best performance in 3 of 4 out-of-sample tasks. To further evaluate the performance of Egret and DRFP, we compared the mean absolute error (MAE) and root mean squared error (RMSE) of the 2 models on the above 11 splits. As shown in Table S4, Egret’s MAE was better than DRFP in 8 of the 11 splits, and Egret achieved better MAE and RMSE than DRFP in 3 of the 4 out-of-sample tasks. In the out-of-sample tasks, the splits were defined by the presence of isoxazole additives that strongly influence reactivity. Therefore, the model had to appropriately extrapolate to unseen additives to perform well, indicating that Egret possesses better robustness and generalization capabilities. Figure 4 is the regression graph of Egret on 11 data split methods. It can be observed from Fig. 4A to H that the model has learned to reasonably predict yields even when the training set was markedly smaller (20%). Moreover, the model’s performance gradually improves as the proportion of the training set increases, and the performance difference on the test set decreases with further augmentation of training data. Figure 4I to L demonstrates that in the out-of-sample tests 1 to 4, Egret demonstrates a satisfactory predictive capability for test 1 and test 2, but the prediction performance on test 3 and test 4 needs to be further improved. The performance of Egret was further tested on the 70:30 (train:test) split of the Suzuki–Miyaura dataset. We conducted 10 different random folds and utilized the same hyperparameter optimization method as the Buchwald–Hartwig dataset. The test results, as shown in Table 2, show that Egret achieved the best performance at the dataset. Regarding the 2 USPTO yield datasets, in addition to maintaining the data split consistency with prior works [31,32], we redivided the training, validation, and test sets at a 6:2:2 ratio to validate the model’s stability. Table 2 indicates that Egret achieved the best performance on the 2 datasets under 2 data segmentation approaches, except for the USPTO gram dataset with reference split. The above results confirm that Egret has competitive predictive power in yield prediction on the HTE datasets. Moreover, the impressive performance on the USPTO yield datasets showcases the promising predictive potential of Egret for generic reaction yield datasets.

**Table 1.** *R*^2^ of Egret on the Buchwald–Hartwig dataset and comparison with other methods (boldface indicates the best performance, and the same applies to the following tables)

*R* ^2^	Methods			
	DFT	Yield-BERT	DRFP	Egret
Rand 70/30	0.92	0.95 ± 0.01	**0.95 ± 0.01**	0.94 ± 0.01
Rand 50/50	0.9	0.92 ± 0.01	0.93 ± 0.01	**0.93 ± 0.01**
Rand 30/70	0.85	0.88 ± 0.01	0.89 ± 0.01	**0.89 ± 0.01**
Rand 20/80	0.81	0.86 ± 0.01	**0.87 ± 0.01**	0.86 ± 0.02
Rand 10/90	0.77	0.79 ± 0.02	**0.81 ± 0.01**	0.80 ± 0.01
Rand 5/95	0.68	0.61 ± 0.04	0.73 ± 0.02	**0.73 ± 0.02**
Rand 2.5/97.5	0.59	0.45 ± 0.05	**0.62 ± 0.04**	0.50 ± 0.12
Test 1	0.8	0.84 ± 0.01	0.81 ± 0.01	**0.84 ± 0.01**
Test 2	0.77	0.84 ± 0.03	0.83 ± 0.003	**0.88 ± 0.03**
Test 3	0.64	**0.75 ± 0.04**	0.71 ± 0.001	0.65 ± 0.06
Test 4	0.54	0.49 ± 0.05	0.49 ± 0.004	**0.54 ± 0.06**

**Table 2.** Performance of Egret on the Suzuki–Miyaura dataset and the USPTO datasets and comparison with other methods

Methods	Metric	Dataset				
		Suzuki–Miyaura	USPTO (grams)		USPTO (subgrams)	
		70/30^a^	Reference split^b^	6:2:2	Reference split^c^	6:2:2
Yield-BERT	*R* ^2^	0.81 ± 0.01	0.117	0.112	0.195	0.171
	MAE	0.08 ± 0.003	0.157	0.156	0.196	0.198
	RMSE	0.12 ± 0.004	0.197	0.196	0.239	0.242
DRFP	*R* ^2^	0.85 ± 0.01	**0.130**	0.118	0.197	0.183
	MAE	0.07 ± 0.002	0.156	0.156	0.197	0.198
	RMSE	0.11 ± 0.004	0.195	0.196	0.239	0.240
Egret	*R* ^2^	**0.85 ± 0.01**	0.128	**0.118**	**0.206**	**0.202**
	MAE	**0.07 ± 0.002**	**0.154**	**0.156**	**0.194**	**0.196**
	RMSE	**0.11 ± 0.004**	**0.195**	**0.196**	**0.237**	**0.238**

^a^ Train/test = 70/30, using 10 different random folds, validation set split from training set.

^b^ Train/test = 158,095/39,524, validation set split from training set.

^c^ Train/test = 241,632/60,408, validation set split from training set.

#### Reaxys-MultiCondi-Yield dataset

Compared to the HTE yield datasets and USPTO yield datasets, Reaxys-MultiCondi-Yield offers a broader chemical space and more comprehensive information about reaction conditions, which strives to resemble the real-world scenarios for chemical synthesis. Here, we conducted 2 subtasks on this dataset—regression for continuous yields and multiclassification for yield categories—and compared the performance of Egret with Yield-BERT and DRFP. Unfortunately, as shown in Table 3, although we tried different dataset segmentation methods, none of the models were able to make accurate predictions on the Reaxys-MultiCondi-Yield dataset on the regression task. Even if Egret performed best, its performance was still not satisfactory. Therefore, we explored the influence of modeling strategies on the predictive performance. As described above, the reactions in the Reaxys-MultiCondi-Yield dataset were classified into 12 categories (Fig. 3A). Except for the category resolutions (due to insufficient data), we separately fine-tuned Egret on the training set of each reaction category and conducted testing on their respective test sets (classification modeling). In addition, we also tested Egret, which was fine-tuned on the entire training set of Reaxys-MultiCondi-Yield, on the test set of each reaction category (overall modeling), and compared the performance of these 2 modeling strategies. From Fig. 3B, it can be observed that Egret exhibited varying predictive performance on different reaction categories. However, the overall modeling outperformed the classification modeling on 9 of 11 reaction categories. This indicates that integrating all types of reaction data for training is meaningful, and the model appears to effectively learn deeper and transferable knowledge across different types of chemical reactions.

**Table 3.** Performance of Egret on the Reaxys-MultiCondi-Yield dataset and comparison with other methods

Methods	Regression						Multiclassification
	8:1:1			6:2:2			8:1:1
	*R* ^2^	MAE	RMSE	*R* ^2^	MAE	RMSE	Accuracy
Yield-BERT	0.1439	0.1317	0.1769	0.1249	0.1363	0.1782	0.5836
DRFP	0.1458	0.1306	0.1767	0.1262	0.1320	0.1780	0.5849
Egret	**0.1529**	**0.1300**	**0.1760**	**0.1429**	**0.1306**	**0.1764**	**0.6016**

Previous studies have shown that in generic reaction yield datasets, although the reactions of the same category exhibit consistent yield trends, different subreactions within the same category often have different yield values [31]. This results in excessive local noise, which affects the performance of the yield prediction models. To fully consider the influence of reaction conditions on reaction yield, the Reaxys-MultiCondi-Yield dataset was constructed with 11,831 reaction groups. Each reaction group consists of a series of reactions with identical reactants and products but varying yields due to different reaction conditions. Inevitably, in addition to the inherent noise in the reaction data, this also increases the local noise and affects the performance of continuous yield regression predictions. In contrast to modeling HTE reaction yield data (stringent control of variables), the task of modeling general reaction yield data is substantially more complex because of not only the characteristics of the recorded reaction data but also unrecorded factors such as laboratory conditions, instrument precision, and differences in personnel operations. These variables substantially increase the difficulty of the modeling process, leading to a pronounced disparity in the model’s predictive performance on HTE reaction data versus general reaction data.

In fact, the yield of a reaction is not an exact constant in the chemistry laboratory. It is influenced not only by the reaction conditions but also by various factors such as the laboratory environment, the operator’s technical skills, and the purity of the reactants. As a result, the yield often fluctuates within a certain range. Therefore, predicting a reasonable yield interval for chemical reactions is also of great significance for guiding chemical synthesis. Here, on the basis of the common yield ranges in chemistry: 80% to 100%, 50% to 80%, 30% to 50%, and 0 to 30%, we categorized the yield data from Reaxys-MultiCondi-Yield into 4 categories: high, medium, low, and extremely low. We evaluated the performance of the multiclass-Egret (MC-Egret) on this dataset and compared it with Yield-BERT and DRFP. As shown in Table 3, MC-Egret achieved a prediction accuracy of 60.16%, higher than both BERT and DRFP. This indicates that Egret exhibited the best predictive performance on the Reaxys-MultiCondi-Yield dataset.

### Contrastive learning pretraining task can enhance the model’s sensitivity to reaction conditions

**Fig. 5.**
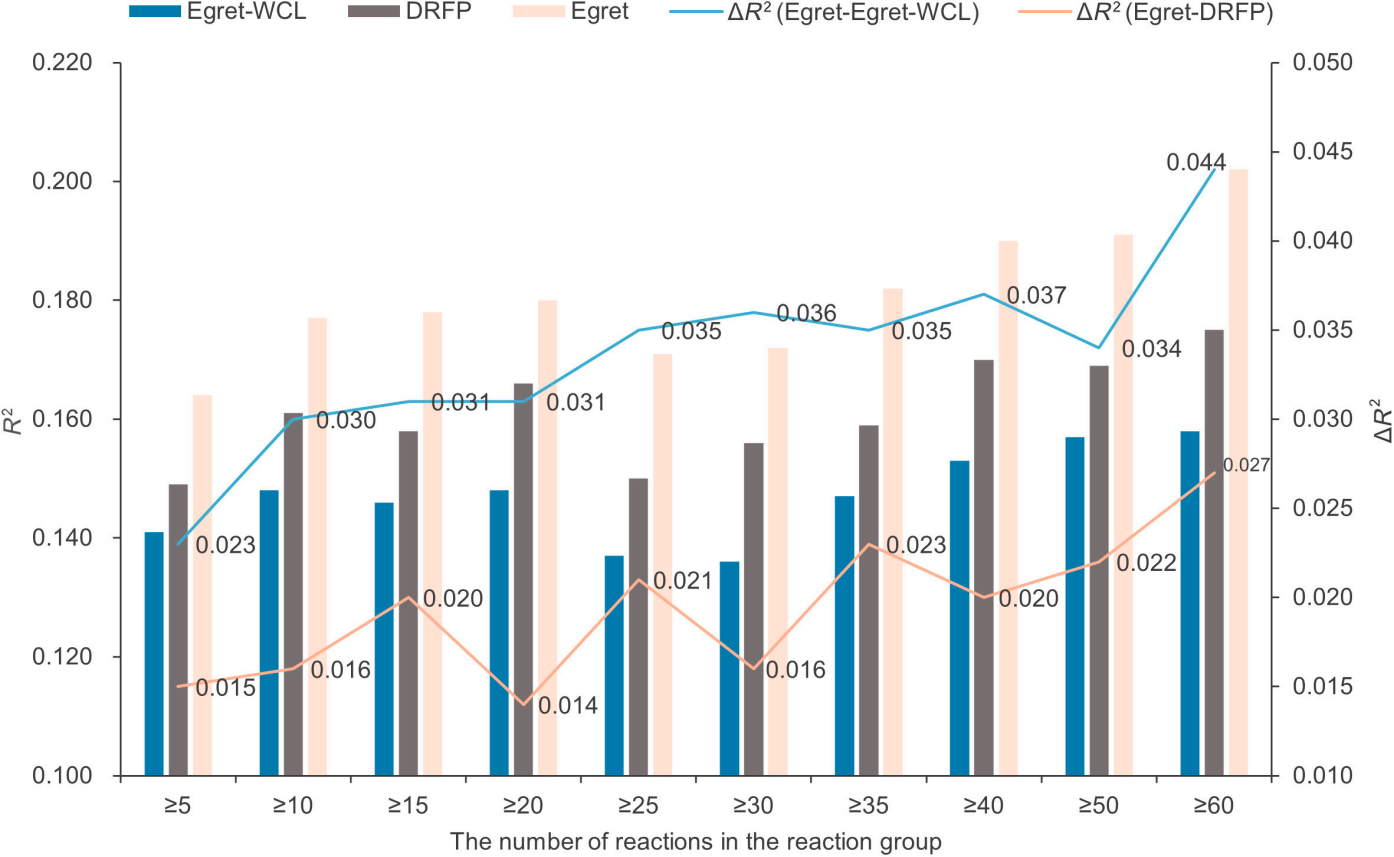
Visualization of the effects of the reaction-condition-based comparative learning task.

As mentioned above, the effectiveness of our pretraining framework has been proven. Next, we explored the role of the contrastive learning pretraining task, for which we constructed an Egret without the contrastive learning pretraining task (Egret-WCL) for fair comparison. We collected the reaction IDs of the reaction groups in the Reaxys-MultiCondi-Yield training set that have more than 5 reactions. On the basis of these reaction IDs, we selected their corresponding reactions from the Reaxys-MultiCondi-Yield test set to create a test set for the reaction groups with more than 5 reactions. Following the same approach, we collected the test sets for the reaction groups with more than 10, 15, 20, 25, 30, 35, 40, 50, and 60 reactions, respectively. In addition, we evaluated the yield prediction performance of Egret and Egret-WCL, which were fine-tuned on the Reaxys-MultiCondi-Yield training set, on the test sets of the aforementioned reaction groups. At the same time, we also measured the performance of DRFP on the test sets of these reaction groups. The results are shown in Fig. 5, and we can see that Egret outperformed Egret-WCL and DRFP in the test sets of different reaction groups. These test sets contain a substantial number of reactions with varying yields due to different reaction conditions. This demonstrates that the contrastive learning pretraining based on reaction conditions effectively enhances the model’s sensitivity to reaction conditions. Egret excels in distinguishing reactions with identical reactants and products but differing reaction conditions, and it also exhibits a better understanding of different forms of the same reaction SMILES. Furthermore, by observing the magnitude of performance improvement, we found that as the number of reactions in the reaction group increases, Egret exhibits a more significant improvement in performance compared to Egret-WCL and DRFP. This indicates that when the dataset has a more comprehensive sampling of reaction-condition-yield combinations for the same reaction, Egret captures a finer characterization of subtle differences among these cases featuring the same reactions under different conditions. This not only further enhances the model’s performance in predicting yields but also indicates the exceptional potential of Egret in handling large-scale, high-quality reaction yield datasets.

### Generic yield prediction model enables retrosynthetic planning tool to prioritize high-yield synthetic routes

**Fig. 6.**
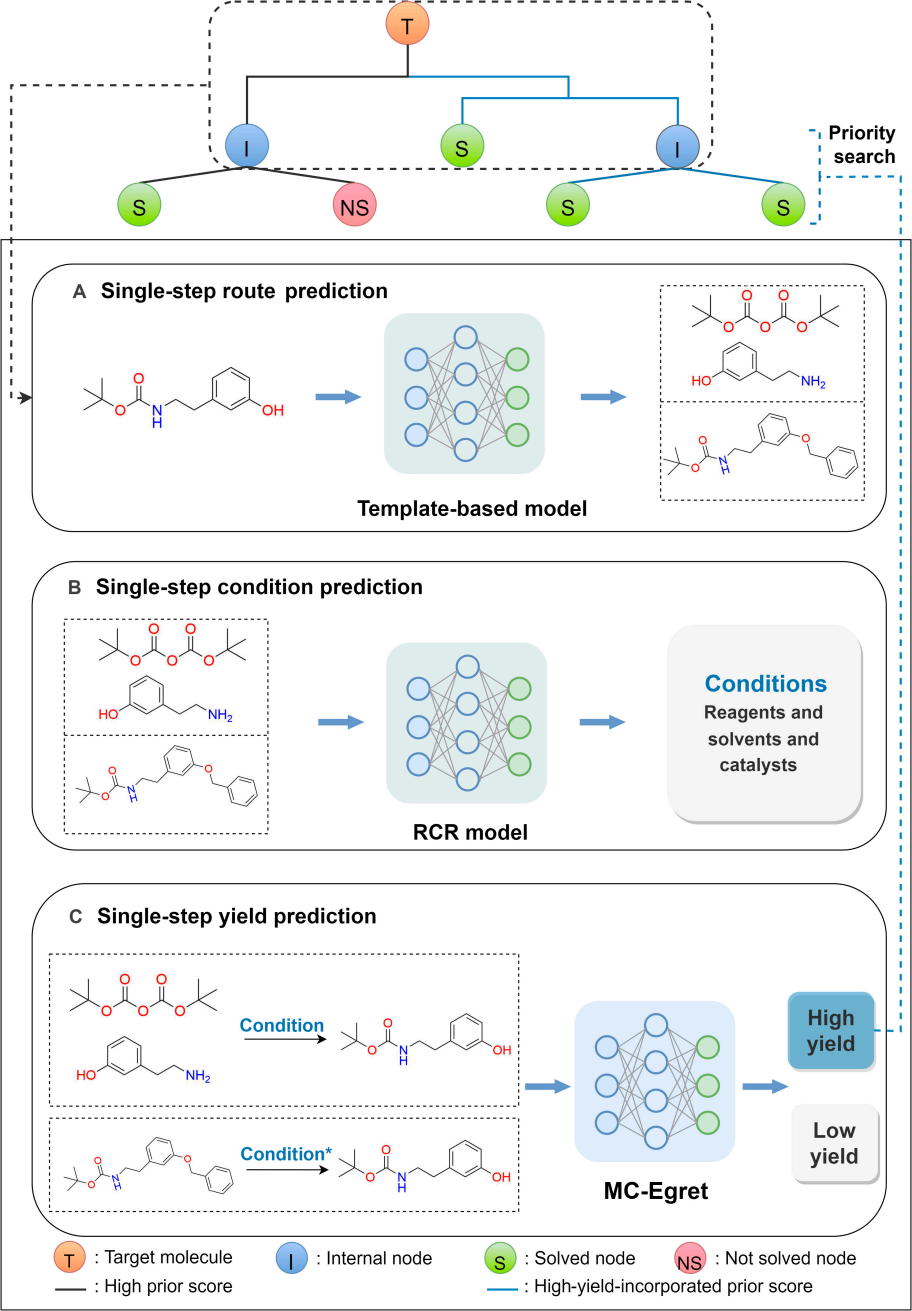
Workflow of the multistep retrosynthetic pipeline embedded with MC-Egret. (A) Single-step route prediction; (B) single-step reaction condition prediction; (C) single-step reaction yield prediction.

The generic yield prediction model, established on the basis of a generic reaction yield dataset, thoroughly learns the relationship between reaction conditions and yields. It possesses the capability to predict yields or yield intervals for any given reaction, without being constrained by specific reaction types. To investigate the application potential of the generic yield prediction model, we proposed a new scoring that incorporates the yield prediction model into a synthetic planning tool and investigated the effects of yield-augmented scoring on synthesis planning. As shown in Fig. 6, we incorporated the MC-Egret trained on Reaxys-MultiCondi-Yield and the reaction condition recommendation model developed by Gao et al. [40] into AiZynthFinder (AZ), a multistep retrosynthesis tool developed by Genheden et al. [11]. MC-Egret was used as part of the prior scores of reaction templates in AZ’s single-step predictions. For an input target molecule, the process begins by utilizing the template-based model within AZ to predict the single-step routes for it (Fig. 6A). Subsequently, the reaction condition recommendation model is used to recommend the reaction conditions for each predicted single-step route (Fig. 6B). The single-step reaction SMILES, which includes the predicted reaction conditions, is then fed into MC-Egret (Fig. 6C). In addition, in the subsequent route prediction, priority is given to expanding molecular nodes within single-step routes that have higher-yield-incorporated prior score.

**Fig. 7.**
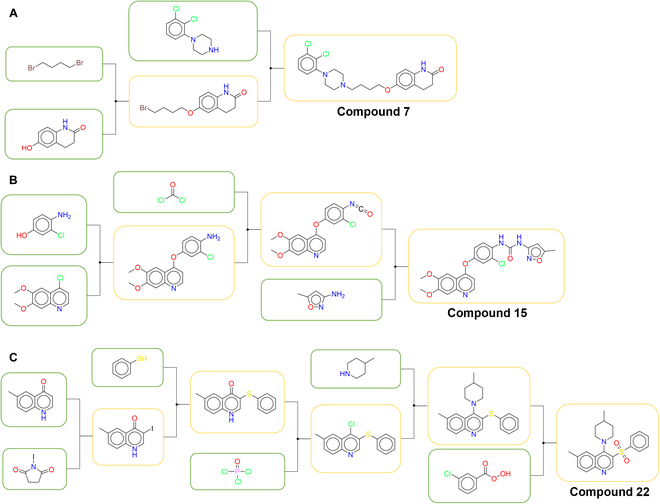
The optimal synthesis routes for compounds 7, 15, and 22.

We randomly selected 50 bioactive small molecules from the PubChem database and conducted the tests both on the original version of AZ and the AZ embedded with MC-Egret (**AZ-Egret**), and we set a max depth of 6, a search time of 120 s, and an iteration of 300 rounds. As shown in Table 4, we selected the compounds that were successfully solved by both methods, and then we found the optimal (higher-yield) synthetic routes for these compounds by diligently consulting the literature. In Table 4, “step” indicates the number of the synthesis steps for the target molecule, while “recommended number” indicates the number of the optimal synthetic route planned by synthesis planning methods. It can be seen that when both AZ-Egret and AZ successfully found the optimal synthesis routes for target molecules, in most cases, the first route planned by AZ-Egret is the optimal synthetic route, while AZ is not. For instance, the optimal synthetic route of compound **7** [50] reported in the literature is shown in Fig. 7A, and the recommended numbers of AZ-Egret and AZ are 1 and 4, respectively. Furthermore, for compounds **13**, **14**, **15**, **17**, **21**, and **22**, only AZ-Egret successfully found the literature-supported synthesis pathways for them. Figure 7B and C is the optimal synthetic routes of compounds **15** [51,52] and **22** [53–55], respectively. In fact, the integration of the yield prediction model extends the search time for single-step prediction. Therefore, within the same search time, the average number of the search iterations of AZ is more than that of AZ-Egret. However, AZ-Egret can still preferentially plan superior synthesis routes for the target molecules with relatively fewer search iterations. The above results indicate that yield-incorporated scoring can facilitate synthesis planning tools to recommend higher-yield and more reasonable synthesis routes for target compounds. Moreover, we have further discussed the diversity and synthetic complexity of the compounds in Table 4 in Section S4, to gain a more comprehensive understanding of the capabilities and characteristics of AZ-Egret. The details about the AZ-Egret and AZ’s planned synthetic routes for the 50 test molecules can be found at https://github.com/xiaodanyin/yield-score-analysis/tree/main/aizynthfinder/aizynthfinder/interfaces.

**Table 4.** Optimal route analysis for compounds for which both AZ and AZ-Egret found synthetic routes

Compound	Recommended number		
	Step	AZ	AZ-Egret
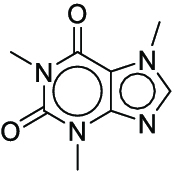 Cn1c(=O)c2c(ncn2C)n(C)c1=O (**1**)	1	1	4
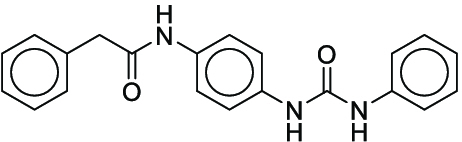 O=C(Cc1ccccc1)Nc1ccc(NC(=O)Nc2ccccc2)cc1 (**2**)	1	2	1
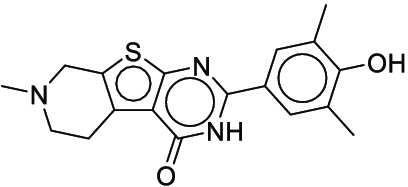 Cc1cc(-c2nc3sc4c(c3c(=O)[nH]2)CCN(C)C4)cc(C)c1O (**3**)	1	2	1
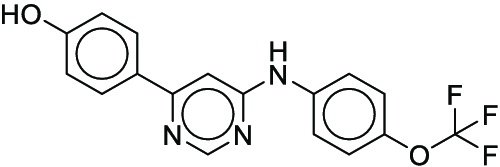 Oc1ccc(-c2cc(Nc3ccc(OC(F)(F)F)cc3)ncn2)cc1 (**4**)	2	5	1
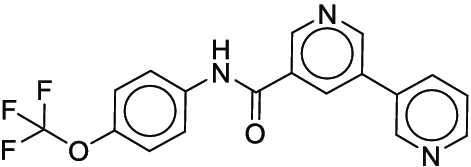 O=C(Nc1ccc(OC(F)(F)F)cc1)c1cncc(-c2cccnc2)c1 (**5**)	2	8	1
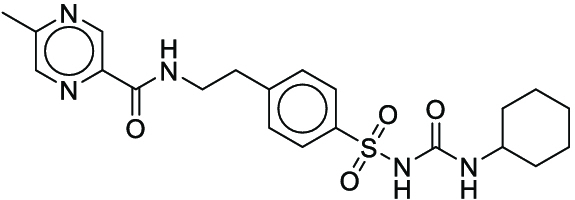 Cc1cnc(C(=O)NCCc2ccc(S(=O)(=O)NC(=O)NC3CCCCC3)cc2)cn1 (**6**)	2	2	1
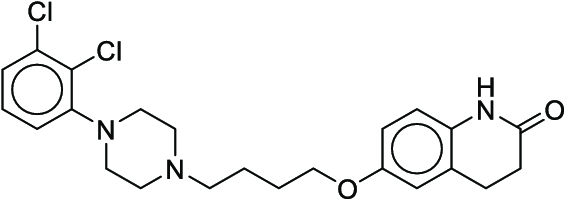 O=C1CCc2cc(OCCCCN3CCN(c4cccc(Cl)c4Cl)CC3)ccc2N1 (**7**)	2	4	1
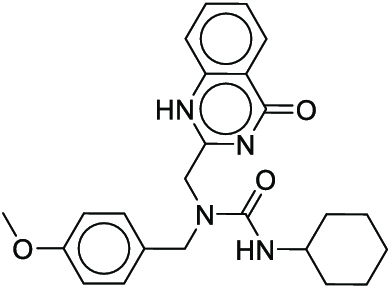 COc1ccc(CN(Cc2nc(=O)c3ccccc3[nH]2)C(=O)NC2CCCCC2)cc1 (**8**)	2	3	1
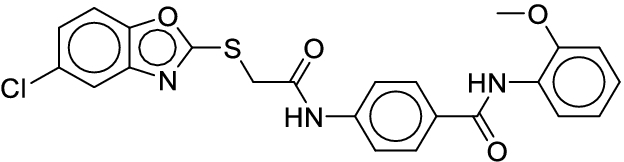 COc1ccccc1NC(=O)c1ccc(NC(=O)CSc2nc3cc(Cl)ccc3o2)cc1 (**9**)	2	3	1
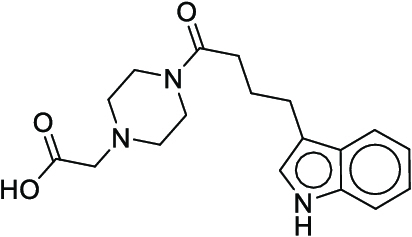 O=C(O)CN1CCN(C(=O)CCCc2c[nH]c3ccccc23)CC1 (**10**)	2	2	1
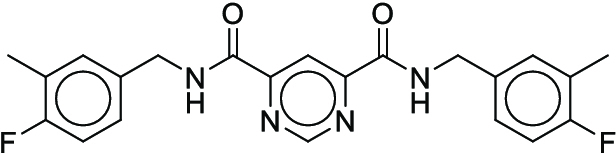 Cc1cc(CNC(=O)c2cc(C(=O)NCc3ccc(F)c(C)c3)ncn2)ccc1F (**11**)	2	4	1
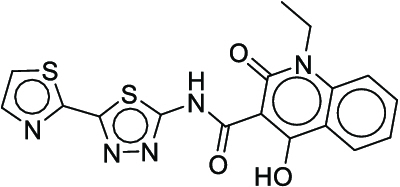 CCn1c(=O)c(C(=O)Nc2nnc(-c3nccs3)s2)c(O)c2ccccc21 (**12**)	2	5	1
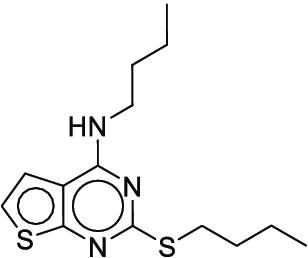 CCCCNc1nc(SCCCC)nc2sccc12 (**13**)	3	-	1
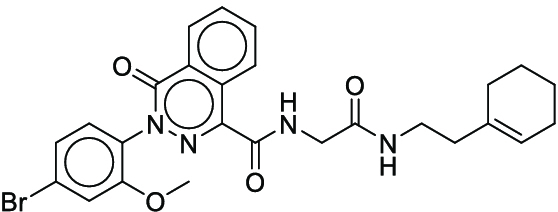 COc1cc(Br)ccc1-n1nc(C(=O)NCC(=O)NCCC2=CCCCC2)c2ccccc2c1=O (**14**)	3	-	1
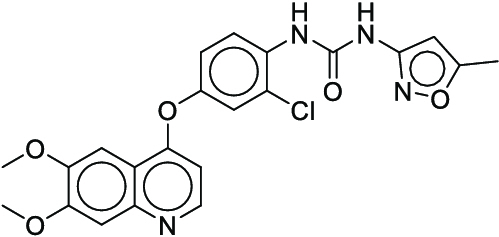 COc1cc2nccc(Oc3ccc(NC(=O)Nc4cc(C)on4)c(Cl)c3)c2cc1OC (**15**)	3	-	1
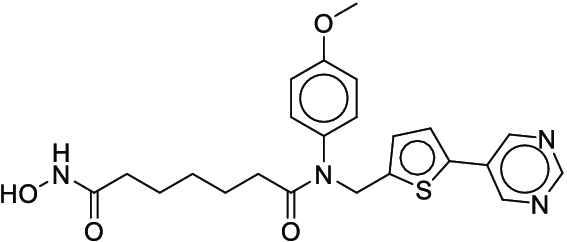 COc1ccc(N(Cc2ccc(-c3cncnc3)s2)C(=O)CCCCCC(=O)NO)cc1 (**16**)	3	2	1
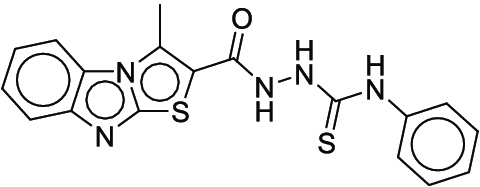 Cc1c(C(=O)NNC(=S)Nc2ccccc2)sc2nc3ccccc3n12 (**17**)	3	-	1
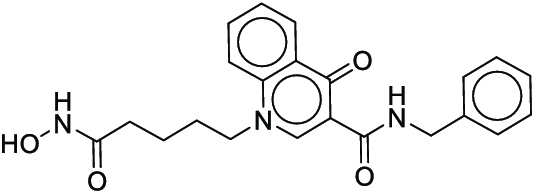 O=C(CCCCn1cc(C(=O)NCc2ccccc2)c(=O)c2ccccc21)NO (**18**)	4	3	1
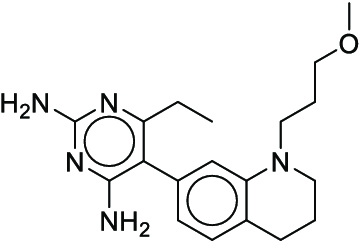 CCc1nc(N)nc(N)c1-c1ccc2c(c1)N(CCCOC)CCC2 (**19**)	4	7	1
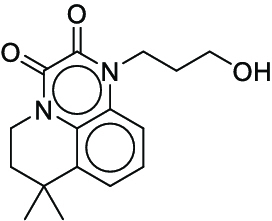 CC1(C)CCn2c(=O)c(=O)n(CCCO)c3cccc1c32 (**20**)	4	4	1
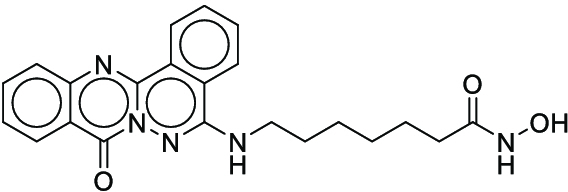 O=C(CCCCCCNc1nn2c(=O)c3ccccc3nc2c2ccccc12)NO (**21**)	4	-	1
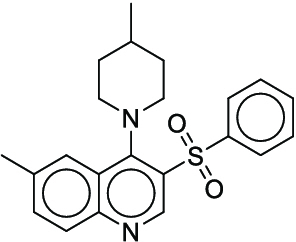 Cc1ccc2ncc(S(=O)(=O)c3ccccc3)c(N3CCC(C)CC3)c2c1 (**22**)	5	-	1
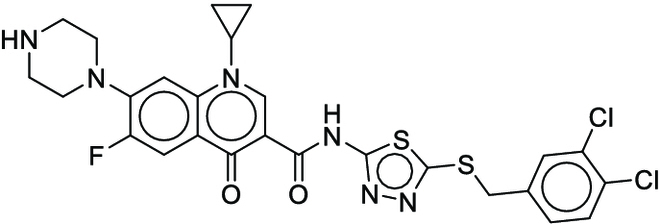 O=C(Nc1nnc(SCc2ccc(Cl)c(Cl)c2)s1)c1cn(C2CC2)c2cc(N3CCNCC3)c(F)cc2c1=O (**23**)	5	5	2

### Meta-learning strategy improves the predictive performance of Egret for reaction types with limited data quantity and low quality

As mentioned earlier, we have experimentally confirmed the positive impact of the generic yield prediction model on synthetic planning. However, the performance of the generic yield prediction model is severely limited because of the lack of a large number of high-quality annotated training data. As described in the “Dataset” section, we utilized a high-accuracy reaction classifier trained on the Pistachio dataset to categorize the reaction data in the Reaxys-MultiCondi-Yield dataset into 12 classes. On this basis, we conducted an analysis by reaction category and evaluated the yield prediction accuracy of MC-Egret on each category of the reaction data in the Reaxys-MultiCondi-Yield validation set and found that the model suffers performance drop for the following reaction types: unrecognized (**0**), deprotections (**6**), functional group interconversion (**9**), functional group addition (**10**), and resolutions (**11**). From Fig. 3A, it is obvious that the reaction types **6**, **9**, **10**, and **11** possessed relatively few reaction data. Category **0**, on the other hand, contains data for which the reaction classifier is unable to predict the reaction type. Despite having a larger data volume, this category contains more complex and lower-quality reaction data. This suggests that the current DL-based reaction yield prediction suffers a low-data problem.

**Fig. 8.**
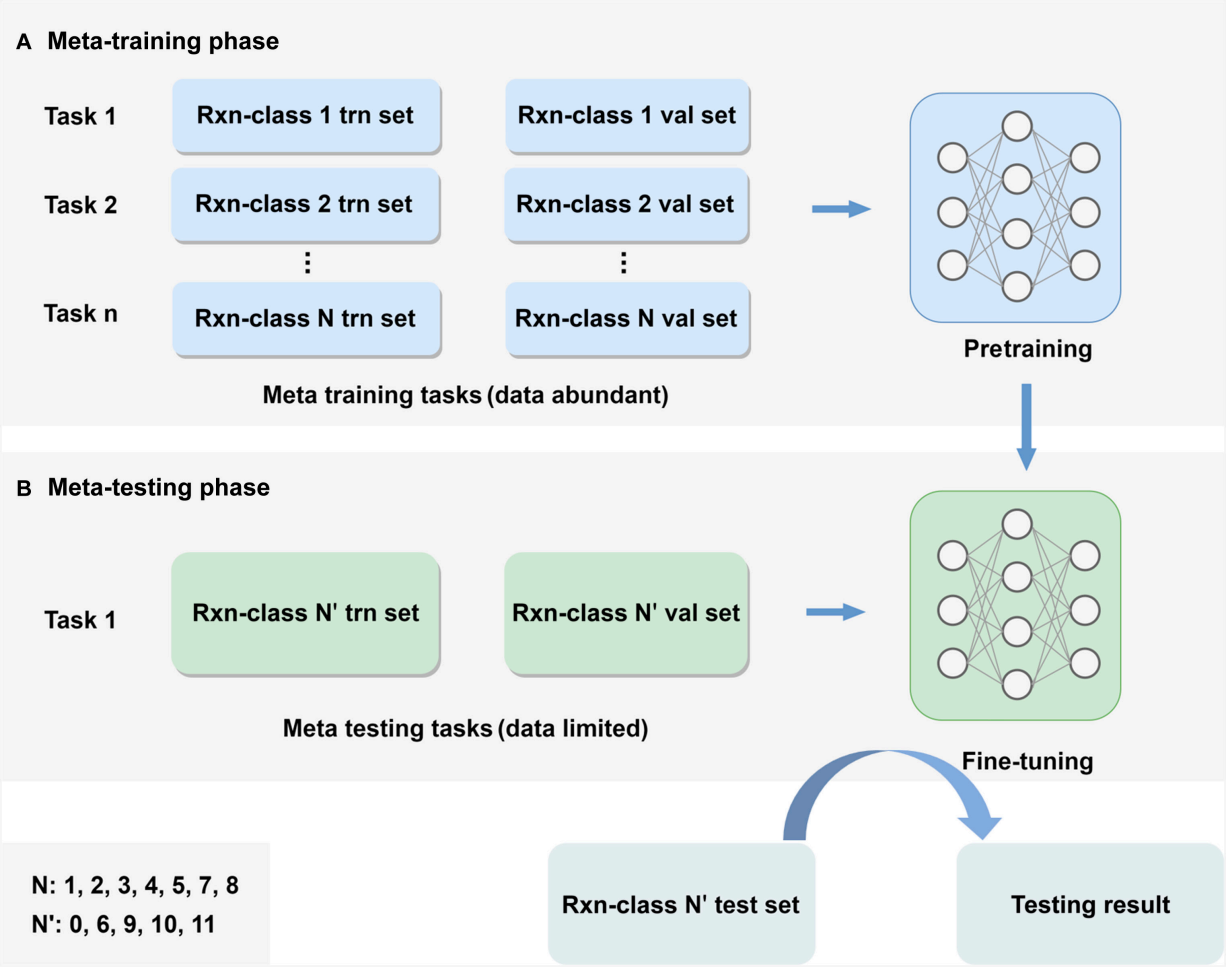
Schematic diagram of Meta-Egret. (A) Meta-training phase; (B) meta-testing phase.

The problem of modeling a small number of training data has been actively studied by the few-shot learning community, and the paradigm of meta-learning has emerged as a noteworthy solution [56–59]. Meta-learning is an advanced machine learning method designed to enable algorithms to optimize and improve the learning process for new tasks through previous learning experiences. It enables models to quickly adapt to unfamiliar tasks by identifying generalizable knowledge or optimization strategies. This “learning to learn” capability is particularly applicable in scenarios requiring models to rapidly learn new information from limited data, and meta-learning has been successfully applied to many low-data scenarios in pharmaceutical and chemical domains. For instance, Chen et al. [33] proposed MetaRF, a random forest model specifically designed for predicting yields with limited data, which achieved satisfactory performance on HTE datasets. Inspired by this, we introduced Meta-Egret in an attempt to improve the yield prediction accuracy of the model for the reaction types with limited data or lower quality in Reaxys-MultiCondi-Yield. As illustrated in Fig. 8, we used the relatively abundant reaction data from the following 7 categories: heteroatom alkylation and arylation (**1**), acylation and related processes (**2**), C–C bond formation (**3**), heterocycle formation (**4**), protections (**5**), reductions (**7**), and oxidations (**8**) as the meta-training tasks. In addition, the training sets (support sets) and validation sets (query sets) for the meta-training tasks were selected from the training and validation sets of Reaxys-MultiCondi-Yield, respectively. The reaction data from the reaction types **0**, **6**, **9**, **10**, and **11** were used as the separate meta-testing tasks. The training set, validation set, and test set of each meta-testing task were obtained from the training, validation, and test sets of Reaxys-MultiCondi-Yield, respectively. During the meta-training phase, the model has the opportunity to learn an internal feature representation that can be broadly applied to predict the reaction yields for various types of reactions, thereby further optimizing its initial model parameters. Subsequently, in the meta-testing phase, fine-tuning and testing will be conducted on the meta-testing tasks with limited data. Table 5 displays the yield prediction accuracy of MC-Egret and Meta-Egret on the respective test sets for the reaction categories **0**, **6**, **9**, **10**, and **11**. The data in the table indicate that, compared to MC-Egret, Meta-Egret enhances the yield prediction performance for these 5 reaction categories to varying degrees. In particular, there is a significant improvement in performance for the reaction category **11**, with an accuracy increase by 33.33%. It is worth noting that the reaction category **11** has the smallest amount of data, once again confirming the effectiveness of the meta-learning strategy under the few-shot setting. However, Meta-Egret shows the smallest improvement in accuracy for the reaction category **0**, suggesting that data quality is the primary bottleneck limiting the enhancement of model performance. In addition, we analyzed the prediction results of MC-Egret and Meta-Egret on the respective test sets of the aforementioned 5 reaction categories and found that the meta-learning strategy improves the model’s prediction accuracy for the long-tail yield labels in these datasets. Figure S3 visualizes the yield distribution of the training set for the reaction category **10**, where it can be observed that the yield data follows a long-tail distribution, with the long-tail data including the reaction data with the “low” and “extremely low” yield labels. On the test set of the reaction category **10**, MC-Egret lacked the ability to predict the “low” yield label (accuracy is 0), but Meta-Egret achieved an accuracy of 15.63% in predicting the “low” yield label. This indicates that the meta-learning strategy also helps alleviate the problem of poor model performance caused by imbalanced data distribution.

**Table 5.** Accuracy of MC-Egret and Meta-Egret on the test sets of the reaction categories 0, 6, 9, 10, and 11

Reaction category	MC-Egret	Meta-Egret
**0**	0.5958	**0.6010**
**6**	0.5596	**0.5927**
**9**	0.5921	**0.6184**
**10**	0.5323	**0.5703**
**11**	0.5556	**0.8889**

## Discussion

On the basis of the well-curated generic reaction yield dataset Reaxys-MultiCondi-Yield, we have successfully developed a generic yield prediction model that can improve the efficiency of synthesis planning. However, it is worth noting that there are still some limitations to our work at present. First, because of the absence of information regarding the time, temperature, and pressure of the chemical reactions in the pretraining dataset Pistachio, the reaction condition information in this work only considers the reagents, solvents, and catalysts. However, the influence of reaction time, temperature, and pressure on reaction yield should not be ignored. Therefore, in future work, we will further incorporate some factors that affect reaction yield, such as time, temperature, and pressure, into the design of yield prediction model. Second, despite the fact that the data in the Reaxys database is sourced from high-quality literature and undergoes rigorous quality control and verification, there are still some unavoidable human errors and a scarcity of reported data on low-yield reactions [60]. Therefore, we also look forward to more unbiased reporting of reaction yield data and the development of more scientific data management tools to promote the advancement of chemical reaction yield prediction tasks.

## Conclusion

In this study, considering the lack of high-quality generic reaction yield datasets, we meticulously curated the Reaxys-MultiCondi-Yield dataset, which contains abundant information on reaction condition and is not limited to a single type of reaction. On the basis of our well-designed pretraining strategies, we proposed a novel yield prediction model called Egret, which achieved the best performance on the Reaxys-MultiCondi-Yield dataset. In addition, Egret exhibited comparable or even superior performance on the 2 HTE yield datasets and 2 USPTO yield datasets when compared to other methods. This indicates the effectiveness of the pretraining strategies of Egret, where contrastive learning enabled the model not only to understand different forms of the same reaction SMILES but also to differentiate the same reaction with varying reaction conditions, thereby enhancing sensitivity to reaction conditions. Consequently, the model could extract chemical reaction features more efficiently and accurately, making it applicable to yield datasets with distinct characteristics. To explore the practical value of the generic yield prediction model, we embedded the generic yield prediction model trained on the Reaxys-MultiCondi-Yield dataset into a multistep retrosynthetic tool. The results demonstrated that yield-incorporated scoring can facilitate synthetic planning tools to prioritize the recommendation of literature-supported reaction pathways with higher yields for target molecules. Furthermore, we further improved the yield prediction accuracy for reaction types with limited data and lower data quality in the Reaxys-MultiCondi-Yield dataset using meta-learning strategies. Specifically, the accuracy for reaction class 10 has been increased by 33.33%. In the future, as more high-quality reaction yield data become available, we believe that Egret and algorithms inspired by Egret will play crucial roles in the development of a comprehensive solution for the DL-based synthesis planning.

## Methods

### Dataset curation

The details of the processing methods for the pretraining dataset and Reaxys-MultiCondi-Yield dataset can be found in Sections S1.1 and S1.2, respectively.

### Model construction and evaluation

### Pretraining of Egret

This process consists of 2 stages. In the first stage, pretraining task 1 was carried out. After the model’s embedding capability for chemical reactions was sufficiently optimized, the second stage of pretraining began. At this stage, both pretraining tasks were performed simultaneously. The 2 phases were pretrained for 50 and 10 epochs, respectively. The transformer encoder used in Egret has 12 layers, each layer contains 4 attention heads, and the hidden size is 256. All codes were implemented in the Python 3.7 environment, the RDKit [61] cheminformatics toolkit was used for data processing, and the model was developed based on the PyTorch library.

#### Fine-tuning of Egret

The DL model’s workflow can be broadly divided into 2 key parts: autonomous feature extraction and downstream task predictions. Egret has learned how to extract the reaction features from the reaction SMILES through pretraining. Then, we kept the same framework to load all the encoding layers in the pretraining model and retrained feedforward neural networks for regression or classification on specific yield prediction datasets.

#### Model evaluation

To evaluate the performance of Egret on 4 benchmark datasets, we compared its performance with the benchmark methods reported previously on these datasets. For the Buchwald–Hartwig dataset, DFT [26], Yield-BERT [31], and DRFP [32] were used as the baselines, and Yield-BERT and DRFP were used as the baselines for the Suzuki–Miyaura dataset and the 2 USPTO yield datasets. Since Yield-BERT and DRFP are the only 2 methods that have been tried on the generic reaction yield datasets (USPTO yield datasets), we used them as the baselines on Reaxys-MultiCondi-Yield too and adopted the regression and multiclassification tasks to evaluate the performance of these methods. The regression tasks were evaluated by the coefficient of determination (*R*^2^), MAE, and RMSE, and the classification tasks were evaluated by the accuracy. Section S2 summarizes the detailed hyperparameters of the Egret, Yield-BERT, and DRFP models and the calculation method of *R*^2^, MAE, RMSE, and accuracy.

### Yield-incorporated synthesis planning analysis

To investigate the application potential of the generic yield prediction model, as shown in Fig. 6, we incorporated MC-Egret into the prior scores of reaction templates in the retrosynthesis planning tool AZ’s single-step predictions to explore the influence of the generic yield prediction model on retrosynthesis planning. The details of how to incorporate MC-Egret into AZ can be found in Section S3.

### Code availability

All the codes of Egret are available at https://github.com/xiaodanyin/Egret, and the code of Egret-incorporated synthesis planning analysis can be found at https://github.com/xiaodanyin/yield-score-analysis.

## Data Availability

Benchmark datasets are available at https://github.com/xiaodanyin/Egret/tree/main/dataset, and we provide the “Reaction ID” and “Links to Reaxys” for all the reactions in Reaxys-MultiCondi-Yield. On the basis of this information, these reactions can be exported from Reaxys to reproduce the dataset. Pistachio [43] dataset can be obtained from NextMove software.

## Supplementary Materials

Supplementary 1 Sections S1 to S5 Figs. S1 to S3 Tables S1 to S5
