# Supplementary material for: Enhancing Generic Reaction Yield Prediction through Reaction Condition-Based Contrastive Learning
Source: Research (Wash D C). 2024 Jan 12;7:0292. doi: 10.34133/research.0292 (PMC10777739; doi:10.34133/research.0292)
Supplement: Supplementary 1 — Sections S1 to S5 Figs. S1 to S3 Tables S1 to S5 [file research.0292.f1.docx]

Supplementary Information for “**Enhancing Generic Reaction Yield Prediction through Reaction Condition-Based Contrastive Learning**”

Xiaodan Yin ^1,4, #^, Chang-Yu Hsieh ^2, #, *^, Xiaorui Wang ^1,4^, Zhenxing Wu ^2,4^, Qing Ye ^2,4^, Honglei Bao^1^, Yafeng Deng ^4^, Hongming Chen ^5^, Pei Luo ^1^, Huanxiang Liu ^3^,

Tingjun Hou ^2, *^, Xiaojun Yao ^3, *^

^1^ Dr. Neher’s Biophysics Laboratory for Innovative Drug Discovery, State Key Laboratory of Quality Research in Chinese Medicine, Macau Institute for Applied Research in Medicine and Health, Macau University of Science and Technology, Macao, 999078, China.

^2^ Innovation Institute for Artificial Intelligence in Medicine of Zhejiang University, College of Pharmaceutical Sciences, Zhejiang University, Hangzhou, 310058, China.

^3^ Faculty of Applied Sciences, Macao Polytechnic University, Macao, 999078, China.

^4^ CarbonSilicon AI Technology Co., Ltd, Hangzhou, Zhejiang 310018, China.

^5^ Center of Chemistry and Chemical Biology, Guangzhou Regenerative Medicine and Health Guangdong Laboratory, Guangzhou 510530, China.

^#^ These authors contributed equally to this work.

^*^**Corresponding authors:**

**Xiaojun Yao**

**E-mail:** xjyao@mpu.edu.mo.

**Tingjun Hou**

**E-mail:** tingjunhou@zju.edu.cn.

**Chang-Yu Hsieh**

**E-mail:** kimhsieh@zju.edu.cn.

**1. Dataset Curation**

**1.1 Pre-training dataset**

Reaction data in Pistachio were used for the pre-training of Egret. The datasets used in pre-training tasks 1 and 2 are Pistachio-Pretraining and Aug-Pistachio-Pretraining respectively, which contain approximately 1.8 million and 7.3 million reaction SMILES. We curated the Pistachio-Pretraining (step1-3) and Aug-Pistachio-Pretraining (step1-5) from the pistachio dataset by the following steps:

1. Deduplication of the original reaction SMILES in the Pistachio dataset, and 2,945,919 data were obtained.
2. Canonicalizing the original reaction SMILES, collecting the original annotated reaction conditions, and removing duplicate data, a total of 1,836,316 data were obtained.
3. Using rxnmapper to reassign atom maps to canonical reaction SMILES, all compounds not participating in product atom contributions were classified as reaction conditions, which were merged with previously collected reaction conditions. Then incorporated the combined reaction conditions into the remaining reaction SMILES (reactants>>product) to obtain the full-reaction SMILES (reactants.conditions>>product), then canonicalized the full-reaction SMILES, and finally removed duplicate data to obtain 1,834,798 pieces of reaction data.
4. Since the Pistachio dataset did not specify the reagents, solvents, and catalysts in the reaction conditions, we manually managed them based on knowledge of chemical reactions. Within the chemical space of reagents, solvents, and catalysts, we randomly replaced the reagents, solvents, and catalysts of each full-reaction once, resulting in a total of 3,669,596 data (negative reaction data augmentation).
5. By permutating the reaction SMILES, each full-reaction SMILES generated a different form of reaction SMILES, and 7,339,192 data were obtained (positive reaction data augmentation), and the processing scripts are available at https://github.com/xiaodanyin/Egret/tree/main/preprocess_script/pretrain_data.

**1.2** **Reaxys-MultiCondi-Yield**

We choose chemical reactions that appeared in the total synthesis literatures as the starting point for cleaning, and the dataset curation steps are as follows:

1. Initially, we collected approximately 17,000 fully synthetic literatures and extracted their DOIs.
2. Leveraging these DOIs, we queried the Reaxys database and obtained 584,061 raw reaction data.
3. Only the data for single-step reactions were selected, half-reactions, reactions with empty yields, and yields that did not match the products were deleted.
4. Canonicalizing the original reaction SMILES, and duplicates were eliminated based on canonical reaction SMILES (reactants>>product), reagents, solvents, catalysts, and yield information. Furthermore, reaction groups containing only one reaction data entry were excluded.
5. The reagents, solvents and catalysts in the dataset were converted from chemical names to SMILES by ChemDraw transformation and network queries. Reaction data whose conditions couldn't be completely converted were removed.
6. Incorporating the reaction conditions (reagents, solvents, catalysts) into the previous canonical reaction SMILES to obtain the full-reaction SMILES (reactants.conditions>>product), and further removed duplicate data to obtain the cleaned Reaxys-MultiCondi-Yield dataset, and the detailed processing scripts are available at https://github.com/xiaodanyin/Egret/tree/main/preprocess_script/reaxys_data. And we provide the "Reaction ID" and "Links to Reaxys" for all the reactions in Reaxys-MultiCondi-Yield (https://github.com/xiaodanyin/Egret/tree/main/dataset/source_dataset/Reaxys-MultiCondi-Yield), based on this information, these reactions can be exported from Reaxys to reproduce the dataset.

**1.3 Visual analysis of the Reaxys-MultiCondi-Yield dataset by reaction type**

To assist readers in better understanding the composition of the Reaxys-MultiCondi-Yield dataset, in addition to visualizing the proportion of various type reaction data contained within it (**Figure 3A in the manuscript**), we further employed the high-dimensional data visualization tool TMAP^1^ to visualize the reaction data of this dataset based on reaction fingerprints. **Figure S1** presents a reaction atlas of the Reaxys-MultiCondi-Yield dataset, where each point represents a chemical reaction, and different colors denote different reaction types. Clusters of colors indicate the density and distribution of the same types of chemical reactions. From **Figure** **S1**, it is observable that, apart from unrecognized reactions, most reaction types tend to cluster, suggesting that reactions of the same type are similar or related in terms of compound structure or reaction fingerprints.


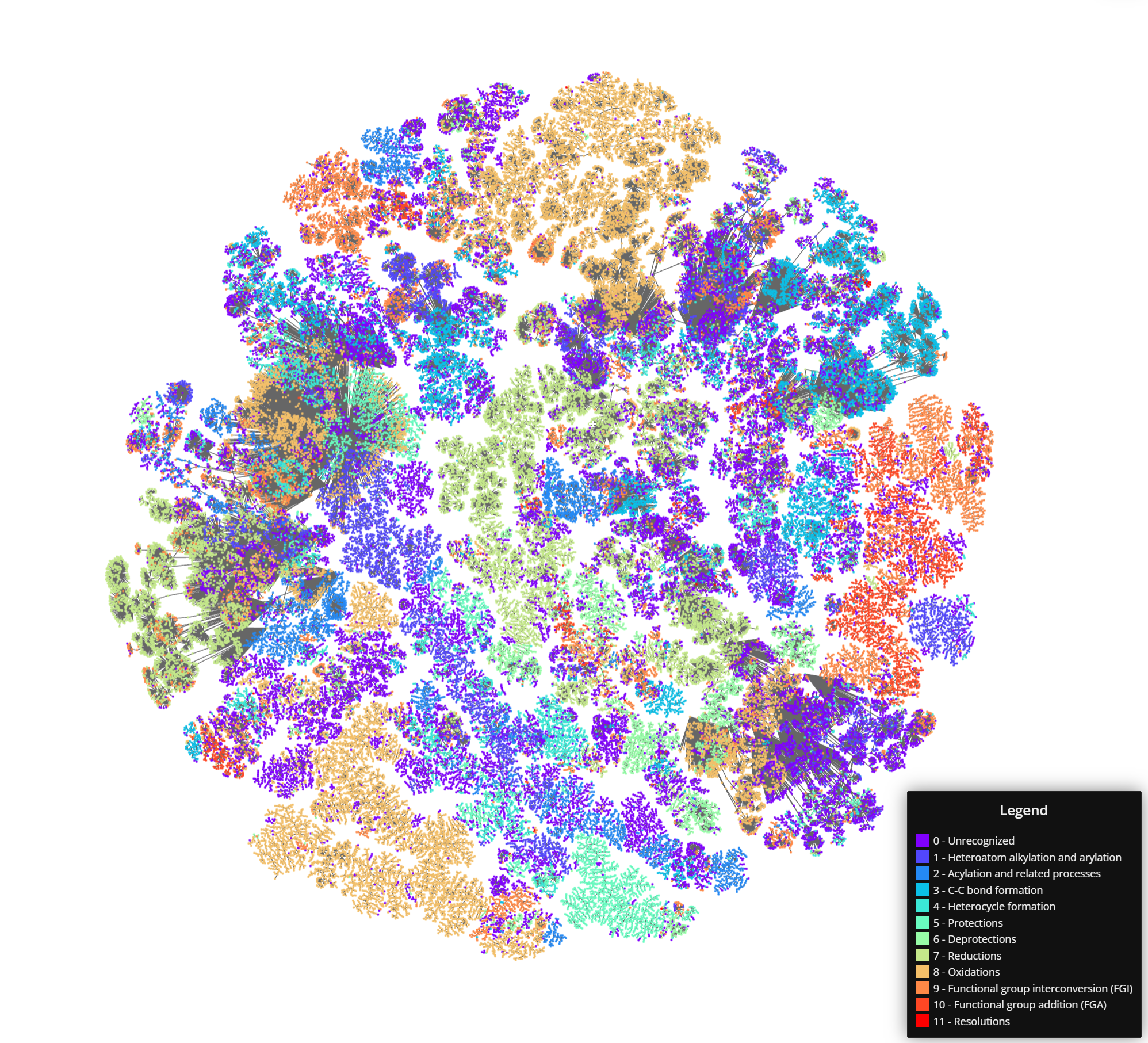


**Figure S1**. Reaction atlas of Reaxys-MultiCondi-Yield dataset.

**1.4 The generation process of positive and negative reaction samples in reaction condition-based contrastive learning**

As shown in the **Figure S2**, for an original reaction, we obtain a negative counterpart through negative augmentation by replacing its reaction conditions. Specifically, this involves randomly substituting the reagents, solvents, and catalysts of the original reaction within the chemical space of reagents, solvents, and catalysts. By contrastive learning, the model learns to distinguish between negative samples, thereby obtaining distinctive feature representations for the same reaction under different reaction conditions. Additionally, SMILES (Simplified Molecular Input Line Entry System), as a method of representing chemical structures with ASCII strings, allows for multiple different SMILES for the same molecule due to the various arrangements and combinations of atoms in chemical molecules. This leads to a variety of valid SMILES forms for a single chemical reaction. In deep learning models, different SMILES strings can lead to variations in the molecular representations learned by the model. Therefore, to obtain a robust representation of chemical reactions, we generate positive reactions by producing an alternatively valid SMILES form for the original reaction SMILES through reaction SMILES permutation. By employing contrastive learning, we aim to maximize the model's representation similarity between positive (i.e. permuted) reaction SMILES and the original reaction SMILES, reducing the uncertainty caused by the diversity of reaction SMILES and thus enhancing the model's understanding of the different SMILES forms for the same reaction.


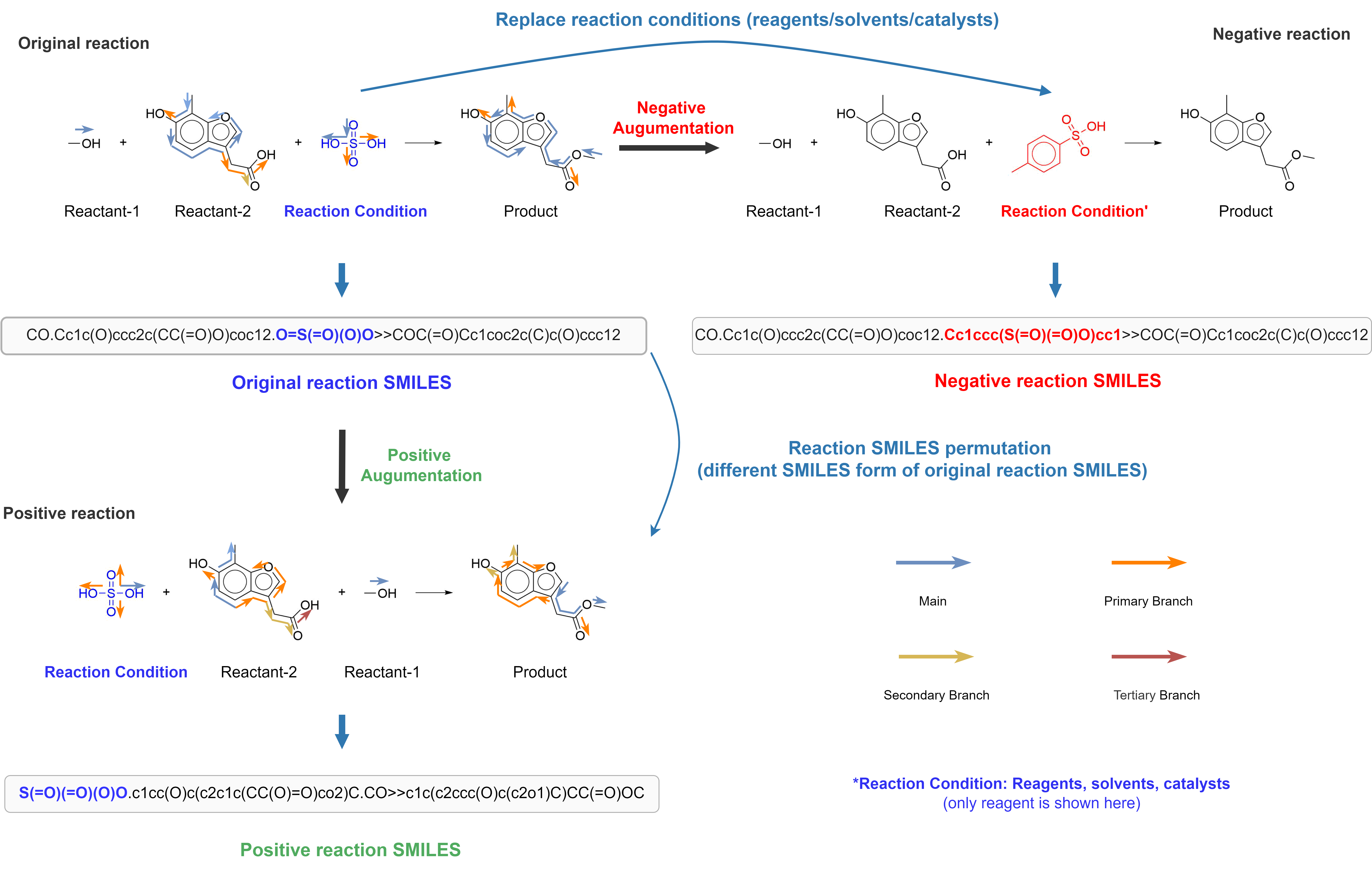


**Figure S2**. The generation process of positive and negative reactions in contrastive learning task.

**2. Model Details**

**2.1 The hyperparameters of Egret pre-training**

**Table S1.** Egret pre-training hyperparameters. (The **bold** font indicates the optimal parameters.)

| **Stage** | **Hyperparameter** | **Value** |
| --- | --- | --- |
| Stage 1 | Number of hidden layers | 12 |
|  | Attention heads | 4 |
|  | Embedding size | 256 |
|  | Hidden size | 256 |
|  | Intermediate size | 512 |
|  | Max padding size | 512 |
|  | Learning rate | 2e-04 |
|  | Epochs | 50 |
|  | Dropout rate | 0.1 |
|  | Train batch size | 32 |
|  | Warmup ratio | 0.06 |
|  | Optimizer | AdamW |
| Stage 2 | Number of hidden layers | 12 |
|  | Attention heads | 4 |
|  | Embedding size | 256 |
|  | Hidden size | 256 |
|  | Intermediate size | 512 |
|  | Max padding size | 512 |
|  | Learning rate | 1e-05 |
|  | Epochs | 10 |
|  | Dropout rate | 0.1 |
|  | Train batch size | 36 |
|  | Warmup ratio | 0.03 |
|  | Optimizer | AdamW |
|  | Different_c | **0.02**, 0.05, 1 |

**2.2 The hyperparameters of Egret Fine-tuning**

**2.2.1 Benchmark datasets**

**Table S2.** Egret fine-tuning hyperparameters on the 4 benchmark datasets.

| **Dataset** | **Hyperparameter** | **Value** |
| --- | --- | --- |
| Buchwald-Hartwig dataset | Learning rate | 8.767e-04^a^ |
|  | Dropout rate | 2.988e-03 |
|  | Train batch size | 16 |
|  | Optimizer | AdamW |
| Suzuki-Miyaura dataset | Learning rate | 1.575e-04^a^ |
|  | Dropout rate | 1.476e-02 |
|  | Train batch size | 16 |
|  | Optimizer | AdamW |
| USPTO gram dataset | Learning rate | 7.448e-05^b^ |
|  | Dropout rate | 1.101e-02 |
|  | Train batch size | 16 |
|  | Optimizer | AdamW |
| USPTO sub-gram dataset | Learning rate | 8.474e-05^b^ |
|  | Dropout rate | 8.091e-03 |
|  | Train batch size | 16 |
|  | Optimizer | AdamW |

^a^ Hyperparameter optimization method: Bayesian optimization.

^b^ Hyperparameter optimization method: Grid search.

**2.2.2 Reaxys-MultiCondi-Yield dataset**

**Table S3.** Egret fine-tuning hyperparameters on the Reaxys-MultiCondi-Yield dataset.

| **Model** | **Hyperparameter** | **Value** |
| --- | --- | --- |
| Egret | Learning rate | 1e-04 |
|  | Dropout rate | 0.1 |
|  | Train batch size | 16 |
|  | Optimizer | AdamW |
| Yield-BERT | Learning rate | 1e-04 |
|  | Dropout rate | 0.1 |
|  | Train batch size | 16 |
|  | Optimizer | AdamW |
| DRFP | Learning rate | 0.1 |
|  | n_estimators | 999999 |
|  | max_depth | 12 |
|  | min_child_weight | 6 |
|  | colsample_bytree | 0.6 |
|  | subsample | 0.8 |

**2.3 MAE and RMSE of Egret on the Buchwald-Hartwig dataset and comparison with DRFP**

**Table S4.** MAE and RMSE of Egret on the Buchwald-Hartwig dataset and comparison with DRFP.

| Split | Egret | | DRFP | |
| --- | --- | --- | --- | --- |
|  | MAE | RMSE | MAE | RMSE |
| Rand 70/30 | 0.0447 ± 0.0023 | 0.0661 ± 0.0030 | **0.0403 ± 0.0013** | **0.0608 ± 0.0028** |
| Rand 50/50 | **0.0469 ± 0.0030** | 0.0730 ± 0.0054 | 0.0478 ± 0.0015 | **0.0715 ± 0.0029** |
| Rand 30/70 | **0.0573 ± 0.0024** | 0.0920 ± 0.0050 | 0.0599 ± 0.0021 | **0.0888 ± 0.0038** |
| Rand 20/80 | **0.0651 ± 0.0028** | 0.1034 ± 0.0061 | 0.0669 ± 0.0022 | **0.0981 ± 0.0029** |
| Rand 10/90 | **0.0806 ± 0.0030** | 0.1233 ± 0.0045 | 0.0841 ± 0.0034 | **0.1197 ± 0.0045** |
| Rand 5/95 | **0.0963 ± 0.0050** | **0.1407 ± 0.0061** | 0.1025 ± 0.0042 | 0.1409 ± 0.0050 |
| Rand 2.5/97.5 | 0.1359 ± 0.0177 | 0.1908 ± 0.0223 | **0.1275 ± 0.0083** | **0.1690 ± 0.0084** |
| Test 1 | **0.0697 ± 0.0047** | **0.1103 ± 0.0064** | 0.0816 ± 0.0007 | 0.1199 ± 0.0010 |
| Test 2 | **0.0631 ± 0.0037** | **0.0941 ± 0.0098** | 0.0769 ± 0.0010 | 0.1126 ± 0.0016 |
| Test 3 | 0.1040 ± 0.0071 | 0.1658 ± 0.0133 | **0.0892 ± 0.0006** | **0.1504 ± 0.0006** |
| Test 4 | **0.1237 ± 0.0083** | **0.1788 ± 0.0099** | 0.1242 ± 0.0007 | 0.1876 ± 0.0011 |

**2.4 Calculation method of R^2^, MAE, RMSE**

$$R^{2}=1-\frac{\sum_{i=1}^{n} \left( y_{i}-\hat{y}_{i} \right)^{2}}{\sum_{i=1}^{n} \left( y_{i}-\bar{y} \right)^{2}}$$

$$MAE=\frac{1}{n}\sum_{i=1}^{n} \left| y_{i}-\hat{y}_{i} \right|$$

$$RMSE=\sqrt{\frac{1}{n}\sum_{i=1}^{n} \left( y_{i}-\hat{y}_{i} \right)^{2}}$$

$$Accuracy=\frac{T_{P}+ T_{N}}{T_{P}+T_{N}+F_{P}+F_{N}}$$

Among them, $T_{P}$, $T_{N}$, $F_{P}$, $F_{N}$ represent true positive, true negative, false positive, and false negative, respectively, $n$ represents the number of samples, $y_{i}$ represents the true value of sample, $\hat{y}_{i}$ represents the predicted value of sample, and $\bar{y}$ represents the mean of the true values.

**3. Calculations for incorporating the MC-Egret** **into the AiZynthFinder**

We integrated reaction condition predictor RCR and our generic reaction yield predictor MC-Egret into Aizynthfinder, enhancing its consideration of reaction conditions and yields by improving the prior scores of reaction templates in Aizynthfinder's single-step predictions. The improved workflow of AiZynthfinder's single-step predictions is as follows:

1. The reaction predicted by the core of the single-step retrosynthetic prediction is input into the reaction condition prediction model RCR. For each predicted reaction, ten sets of reaction conditions are predicted. Among the top k predicted results for the target product P, the i-th reaction $rxn_{i}$ has a prior score of $p^{i}$, and the corresponding set of conditions is denoted as ${Conditions}_{rxn_{i}} = \left\{ C_{1}^{rxn_{i}} , C_{2}^{rxn_{i}}\ldots C_{10}^{rxn_{i}} \right\}$. Here, $C_{j}^{rxn_{i}}$ represents the j-th ranked set of reaction conditions for reaction i, and the confidence scores output by the RCR model are denoted as $ConditionScores_{rxn_{i}}=\{S_{1}^{rxn_{i}} , S_{2}^{rxn_{i}}\ldots S_{10}^{rxn_{i}}\}$.
2. The reaction, along with each set of reaction conditions, is input into the MC-Egret to obtain the yield level $Y_{j}^{rxn_{i}}$(ranging from 0 to 3, with 0 representing the highest yield). The yield score for the corresponding reaction is calculated as $Y^{rxn_{i}} = \frac{\sum_{j}^{10} \left( ( 4-Y_{j}^{rxn_{i}})*S_{j}^{rxn_{i}} \right)}{10}$.
3. The prior score that incorporates the consideration of yield is defined as $\hat{p}^{i} =\frac{p^{i}+Y^{rxn_{i}}}{\sum_{i}^{k} (p^{i}+Y^{rxn_{i}})}$. We replace $p^{i}$ with $\hat{p}^{i}$ in the synthesis path planning.

**4. Analysis of the diversity and synthetic complexity of the compounds in Table 4**

To gain a deeper understanding of the capabilities and characteristics of the multi-step retrosynthetic pipeline AZ-Egret, we analyzed the compounds for which AZ-Egret successfully planned relatively high-yield synthetic routes, as described in **Table 4** of the manuscript. We first assessed the molecular diversity of these 23 compounds, with the calculation formula as follows^2^:

$$Diversity=1-\frac{2}{n(n-1)}\underset{X,Y}{\sum}sim(X,Y)$$

Where $n$ is the total number of molecules, and $sim(X,Y)$ represents the Tanimoto similarity between molecules $X$ and $Y$. Molecular diversity is obtained by subtracting the average Tanimoto similarity across all pairs of molecules from 1, with values ranging from 0 to 1, where a high diversity value implies low similarity. Upon calculation, the diversity of the 23 compounds was 0.9014, indicating that the compounds we randomly selected for testing AZ-Egret are structurally diverse with low similarity. This also reflects AZ-Egret's ability to handle the diverse challenges of chemical space, process collections of molecules with high structural diversity, and propose potential high-yield synthetic routes for molecules of various different structures. Next, we used the reaction classifier as described in the manuscript to analyze each single-step reaction in these 23 advantageous synthetic routes (reaction route with relatively high yield) one by one. As shown in **Table S5**, the reactions involved in synthesizing these compounds include all types of reactions that the reaction classifier can predict, except for deprotection reactions. This indicates that the chemical transformations required for the synthesis of these compounds are diverse, and these compounds possess a high level of chemical complexity. It also reflects the capability of AZ-Egret to handle complex chemical changes.

**Table S5**. Analysis of reaction types of the synthetic routes planned by AZ-Egret.

| Compound | Step | Reaction type/Step* | |
| --- | --- | --- | --- |
| Cn1c(=O)c2c(ncn2C)n(C)c1=O **(1)** | 1 | Heteroatom alkylation and arylation |  |
| O=C(Cc1ccccc1)Nc1ccc(NC(=O)Nc2ccccc2)cc1**(2)** | 1 | Acylation and related processes |  |
| Cc1cc(-c2nc3sc4c(c3c(=O)[nH]2)CCN(C)C4)cc(C)c1O**(3)** | 1 | Heterocycle formation |  |
| Oc1ccc(-c2cc(Nc3ccc(OC(F)(F)F)cc3)ncn2)cc1(**4**) | 2 | C-C bond formation;  Heteroatom alkylation and arylation |  |
| O=C(Nc1ccc(OC(F)(F)F)cc1)c1cncc(-c2cccnc2)c1(**5**) | 2 | Acylation and related processes;  Functional group interconversion (FGI) |  |
| Cc1cnc(C(=O)NCCc2ccc(S(=O)(=O)NC(=O)NC3CCCCC3)cc2)cn1(**6**) | 2 | Acylation and related processes;  Acylation and related processes |  |
| O=C1CCc2cc(OCCCCN3CCN(c4cccc(Cl)c4Cl)CC3)ccc2N1  (**7**) | 2 | Heteroatom alkylation and arylation; Heteroatom alkylation and arylation |  |
| COc1ccc(CN(Cc2nc(=O)c3ccccc3[nH]2)C(=O)NC2CCCCC2)cc1**(8)** | 2 | Heteroatom alkylation and arylation; Acylation and related processes |  |
| COc1ccccc1NC(=O)c1ccc(NC(=O)CSc2nc3cc(Cl)ccc3o2)cc1  **(9)** | 2 | Acylation and related processes; Functional group interconversion (FGI) |  |
| O=C(O)CN1CCN(C(=O)CCCc2c[nH]c3ccccc23)CC1 **(10)** | 2 | Deprotections;  Acylation and related processes |  |
| Cc1cc(CNC(=O)c2cc(C(=O)NCc3ccc(F)c(C)c3)ncn2)ccc1F  **(11)** | 2 | Acylation and related processes;  Acylation and related processes |  |
| CCn1c(=O)c(C(=O)Nc2nnc(-c3nccs3)s2)c(O)c2ccccc21(**12**) | 2 | Acylation and related processes; Functional group interconversion (FGI); Heterocycle formation |  |
| CCCCNc1nc(SCCCC)nc2sccc12**(13)** | 3 | Heteroatom alkylation and arylation; Functional group interconversion (FGI); Heteroatom alkylation and arylation |  |
| COc1cc(Br)ccc1-n1nc(C(=O)NCC(=O)NCCC2=CCCCC2)c2ccccc2c1=O  **(14)** | 3 | Acylation and related processes; Deprotections**;**  Unrecognized |  |
| COc1cc2nccc(Oc3ccc(NC(=O)Nc4cc(C)on4)c(Cl)c3)c2cc1OC  **(15)** | 3 | Acylation and related processes;  Functional group interconversion (FGI); Heteroatom alkylation and arylation |  |
| COc1ccc(N(Cc2ccc(-c3cncnc3)s2)C(=O)CCCCCC(=O)NO)cc1**(16)** | 3 | Acylation and related processes;  Acylation and related processes; Unrecognized;  Functional group interconversion (FGI); Heteroatom alkylation and arylation |  |
| Cc1c(C(=O)NNC(=S)Nc2ccccc2)sc2nc3ccccc3n12**(17)** | 3 | Acylation and related processes; Deprotections;  Unrecognized |  |
| O=C(CCCCn1cc(C(=O)NCc2ccccc2)c(=O)c2ccccc21)NO  **(18)** | 4 | Acylation and related processes; Deprotections;  Unrecognized;  Heteroatom alkylation and arylation; Acylation and related processes |  |
| CCc1nc(N)nc(N)c1-c1ccc2c(c1)N(CCCOC)CCC2(**19**) | 4 | C-C bond formation;  Functional group interconversion (FGI); Reductions;  Heteroatom alkylation and arylation |  |
| CC1(C)CCn2c(=O)c(=O)n(CCCO)c3cccc1c32**(20)** | 4 | Heteroatom alkylation and arylation; Unrecognized;  Unrecognized;  Reductions |  |
| O=C(CCCCCCNc1nn2c(=O)c3ccccc3nc2c2ccccc12)NO  **(21)** | 4 | Acylation and related processes; Deprotections;  Unrecognized;  Heteroatom alkylation and arylation; Unrecognized |  |
| Cc1ccc2ncc(S(=O)(=O)c3ccccc3)c(N3CCC(C)CC3)c2c1**(22)** | 5 | Oxidations;  Heteroatom alkylation and arylation; Functional group interconversion (FGI); Heteroatom alkylation and arylation; 'Functional group addition (FGA) |  |
| O=C(Nc1nnc(SCc2ccc(Cl)c(Cl)c2)s1)c1cn(C2CC2)c2cc(N3CCNCC3)c(F)cc2c1=O**(23)** | 5 | Heteroatom alkylation and arylation; Acylation and related processes; Deprotections;  Heteroatom alkylation and arylation; Unrecognized;  Functional group interconversion (FGI); Unrecognized |  |

* When a single step contains two branch reactions, there will be a situation where the number of reaction steps is not equal to the number of reaction types.

**5. Visualization of yield distribution for reaction category 10 training set**


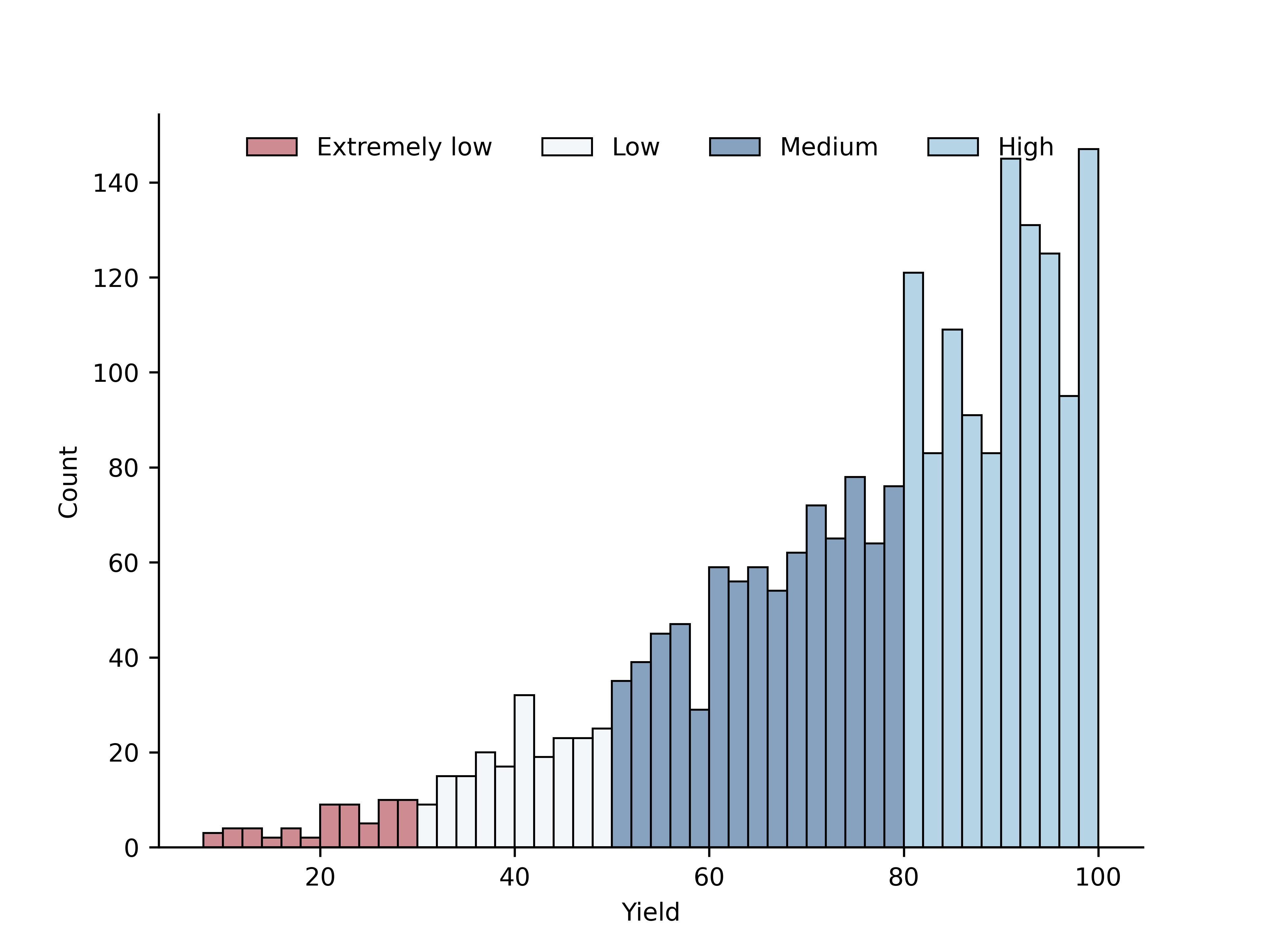


**Figure S3**. Yield distribution of the training set for reaction category 10.

**References**

(1) Probst, D.; Reymond, J.-L. Visualization of Very Large High-Dimensional Data Sets as Minimum Spanning Trees. *J. Cheminform.* **2020**, 12, 1-13.

(2) Wang, J.; Hsieh, C.-Y.; Wang, M.; Wang, X.; Wu, Z.; Jiang, D.; Liao, B.; Zhang, X.; Yang, B.; He, Q. Multi-Constraint Molecular Generation Based on Conditional Transformer, Knowledge Distillation and Reinforcement Learning. *Nat. mach. intell.* **2021**, 3, 914-922.
